# Proteomic and Systems Biology Analysis of Monocytes Exposed to Securinine, a GABA_A_ Receptor Antagonist and Immune Adjuvant

**DOI:** 10.1371/journal.pone.0041278

**Published:** 2012-09-13

**Authors:** Matt Shipman, Kirk Lubick, David Fouchard, Rajani Guram, Paul Grieco, Mark Jutila, Edward A. Dratz

**Affiliations:** 1 Department of Chemistry and Biochemistry, Montana State University, Bozeman, Montana, United States of America; 2 Department of Veterinary Molecular Biology, Montana State University, Bozeman, Montana, United States of America; University of Pittsburgh, United States of America

## Abstract

Securinine, a GABA_A_ receptor antagonist, has been reported to enhance monocyte cell killing of *Coxiella burnetii* without obvious adverse effects *in vivo*. We employed multiplex 2D gel electrophoresis using Zdyes, a new generation of covalently linked fluorescent differential protein detection dyes to analyze changes in the monocyte proteome in response to Securinine. Securinine antagonism of GABA_A_ receptors triggers the activation of p38. We used the differential protein expression results to guide a search of the literature and network analysis software to construct a systems biology model of the effect of Securinine on monocytes. The model suggests that various metabolic modulators (fatty acid binding protein 5, inosine 5′-monophosphate dehydrogenase, and thioredoxin) are at least partially reshaping the metabolic landscape within the monocytes. The actin bundling protein L-plastin, and the Ca^2+^ binding protein S100A4 also appear to have important roles in the immune response stimulated by Securinine. Fatty acid binding protein 5 (FABP5) may be involved in effecting lipid raft composition, inflammation, and hormonal regulation of monocytes, and the model suggests that FABP5 may be a central regulator of metabolism in activated monocytes. The model also suggests that the heat shock proteins have a significant impact on the monocyte immune response. The model provides a framework to guide future investigations into the mechanisms of Securinine action and with elaboration may help guide development of new types of immune adjuvants.

## Introduction

It was recently reported by Lubick, et al, that Securinine enhanced monocytic cell killing of phase II *Coxiella burnetii* [1]. Small molecules, such as Securinine, that activate the innate immune response are adjuvants, and represent an alternative and/or complimentary method to vaccines as a means to protect against infection. Adjuvants can be given either prior to potential risk of exposure to prophylactically fend off infection before it starts, or possibly as part of a treatment regimen after infection. In addition, since adjuvant responses do not depend on specific antigens, an adjuvant can potentially have beneficial therapeutic effects on a wide range of infectious agents, even when no vaccine is available.

Securinine specifically binds to and blocks the GABA binding site on GABA_A_ receptors [2]. GABA_A_ receptors have been shown to be present in both monocytes [3], [4] and T cells and GABA is present in the bloodstream at levels comparable to the levels in the central nervous system (CNS) synapses [5]. GABA_A_ receptors are important inhibitory neurotransmitter receptors in the CNS, and recent reports have suggested that they have a role in modulating the immune response in both monocytes and T cells [4], [5]. We undertook a proteomic and systems biology investigation of the effects of Securinine on Monomac I cells, a cultured monocyte cell line, previously investigated by Lubick et al. [1]. Lubick et al., demonstrated that Securinine modulates the monocyte immune response in a manner distinct from that of Toll-like receptor (TLR) activation, and that Securinine does not trigger TLRs [1]. Our results, as well as evidence from other studies support the conclusion that immune modulation due to Securinine antagonism of GABA_A_ receptors is distinct from immune modulation due to TLR stimulation [6], [7][Shipman, et al., in preparation]. Inhibition of the GABA_A_ receptors by Securinine appears to trigger maturation of the monocytes into macrophages, as well as priming aspects of the immune response, including antigen presentation mechanisms, as described below. Securinine thus represents a novel class of innate immune adjuvant that may have applications in strategies protecting against *C. burnetii* and other pathogens.

## Materials and Methods

### Sample preparation

Monomac I cultures (DSM ACC 252, German Micro-organism and Cell Culture Collection, Braunschweig, Germany) were grown and treated with 25 uM Securinine for 24 hours and harvested as previously described [1] [Shipman et al., submitted]. Harvested cells were lysed on ice in 1 ml aliquots, using a hand-driven, tight-fitting Dounce homogenizer, four rounds of sonication on ice, a freeze thaw cycle to −80°C, and two rounds of sonication on ice [8], [9]. Sonication was performed using fifteen 1 second bursts with 1 second in between each burst (as described in supplementary materials). Samples sat on ice for at least 1 minute between rounds of sonication to decrease temperature and lysis was determined by cell counting in a hemacytometer. Aliquots were subjected to high speed centrifugation for 45 minutes at 100,000×g and 4°C, using a TSL100 ultracentrifuge with a TL-55 rotor (Beckman) to pellet membranes and organelles. The cytosolic supernatant was harvested and the samples were treated as described (Shipman, et al., submitted) and in Methods S1. Like aliquots were pooled, and quantified by the Bradford protein assay (Biorad, item 500-0006, Hercules CA) or stored at −80°C, as described in Methods S1.

### Labeling reactions and separation conditions for multiplex 2DE

100 ug of protein per channel (control and experimental cytosol or membrane samples) were labeled with different colored Zdyes (ZGB, a green fluorophore, and ZBB a blue fluorophore) [10]–[12] and reciprocal color labeling was carried out on duplicate samples, as described in Methods S1. The samples were brought to 5 mg/mL final protein concentration with pH 8.5 labeling buffer containing detergent(s) appropriate for the sample type. Labeling and first dimension IEF separations were performed as described in Methods S1.

Acrylamide gradient gels (9.5–16% for membrane samples and 9.5–18% for soluble fraction samples) were cast using 1.5 mm spacer plates and a GE Healthcare casting chamber as described in Shipman, et al., submitted. Running conditions for the 2^nd^ dimension separation were as described previously (Shipman, et al., submitted). Gels were scanned, using a Typhoon Trio gel scanner (GE Healthcare), at 200 um resolution with laser excitation at 488 nm, 532 nm, and 633 nm and with the PMT voltage set to just below the threshold of pixel saturation.

### Gel analysis

Images were uploaded into the Progenesis software package (v. 2 Nonlinear Dynamics), using the setting for multiple dyes without DIGE structure, as described in a companion paper (Shipman, et al., submitted). Gel spot patterns were aligned using both manual and autovectors and the alignments were manually validated. A mixed linear model statistical analysis of the differentially expressed spots was performed as described in Shipman, et al., (submitted). For the final decision on the differential regulation of protein spots, a p-value from the mixed linear model was required to be <0.05 with an observed power score >0.7.

### Spot picking, in-gel digestion, and MS anaysis

Gels utilized for spot picking and MS analysis were stained with the Blue Silver formulation of Coomassie blue (described in the Methods S1). A spot map was generated from Progenesis analysis, indicating the spots to be picked and a unique label was established for each picked spot. In-gel digestion protocols were adapted from Barry, et al. [13], [14]. Following drying, the gel pieces were rehydrated with modified porcine trypsin solution (Promega) [12.5 ng trypsin/uL in 25 mM NH_4_HCO_3_/10% (v/v) acetonitrile pH 8.0] [13]–[15], using enough solution to cover the pieces with ∼3× their dry volume. Gel pieces were rehydrated on ice for 30 minutes, and excess trypsin solution was removed. The gel pieces were covered with 25 mM NH_4_HCO_3_/10% (v/v) acetonitrile pH 8.0 [15] and incubated overnight at 37°C.

Digests were transferred to Eppendorf tubes containing 10 uL of 50% (v/v) acetonitrile/5% (v/v) formic acid (Fisher Scientific, ACS grade). The peptides were extracted from the gel pieces three times using fresh 50% (v/v) acetonitrile/5% (v/v) formic acid (as described further in Methods S1), and either stored at −80°C or used immediately for MS analysis. Digests were analyzed using an Agilent ChipLC system with a 150 mm separation column (part# G4240-62002) and Agilent XCT Ultra Ion trap mass spectrometer, using: Solvent A [95% (v/v) water (Fisher Scientific, HPLC grade)/5% (v/v) acetonitrile (Fisher Scientific, HPLC grade)/0.1% (v/v) formic acid] and Solvent B [5% (v/v) water/95% (v/v) acetonitrile (HPLC grade)/0.1% (v/v) formic acid]. Completed runs were analyzed using Bruker Daltonics Data Analysis software.

### Bioinformatics

Three bioinformatics tools were used for analysis of the mass spectrometer data, Mascot (licensed in-house at Montana State University) [16] and the X!Hunter and X!Tandem P3 algorithms from www.thegpm.org [17]–[20]. One missed cleavage by trypsin was allowed and the parent ion mass tolerance was set to 0.8 Da. The NCBInr human database was searched in Mascot and an MS/MS tolerance of 0.3 Da was used, with cysteine carbamidomethylation, deamidation and methionine oxidation included as variable parameters for +2 and +3 ions. For both X!Hunter and X!Tandem P3 searches, the human database was searched and default parameters were used, with the exception of the 0.8 Da parent ion mass tolerance. For X!Hunter only +2 charges were searched. Protein identifications that were not keratin or trypsin, and were the highest ranked protein identification that most closely matching the observed molecular weight and pI were accepted. In most cases a single protein had a much more favorable score with all three bioinformatic tools than the second closest ranking protein and the identified proteins had high (16–46%) sequence coverages. A minimum of two unique, statistically significant peptides were required for protein identification from all bioinformatics tools. Mascot protein scores are reported as the sum of peptide scores from all statistically significant peptides.

### Western Blot

MonoMac-1 cells were treated with DMSO, 50 uM securinine, or 20 ug/ml Anisomycin (Sigma) for 30 min, 60 min, or 4 hrs. At the indicated time-points, cells were transferred to tubes and collected by centrifugation for 5 min at 500 g. Supernatant fluids were removed and discarded, and cell pellets were suspended in buffer containing 0.15 m NaCl, 1% sodium deoxycholate, 1% Triton X-100, 0.1% SDS and 10 mM Tris-HCL pH 7.2, and were incubated on ice for 30 min. Samples were then boiled for 5 min before separation on a 10% sodium dodecyl sulfate-PAGE gel. Proteins in the gels were transferred to nitrocellulose, which was then blocked in 10% Blotto (10% w/v non-fat dry milk in PBS) for 1 h at RT. Blots were incubated with the following antibodies diluted 1∶1000 in 0.5% Blotto at RT overnight: anti-phospho p38 MAPK (Thr180/Tyr182) (Cell Signaling), anti-p38 MAPK (Cell Signaling), or anti-GABA_A_ 1 α (Lab Vision). Blots were washed three times with 0.5% Blotto and incubated with HRP-goat anti-rabbit secondary antibody at 1∶5000 in 0.5% Blotto for 2 h at RT. Blots were washed and developed with ECL reagent (GE Healthcare, Piscataway, NJ, USA) and exposed to film for detection of chemiluminescence.

## Results

**Figure 1:**
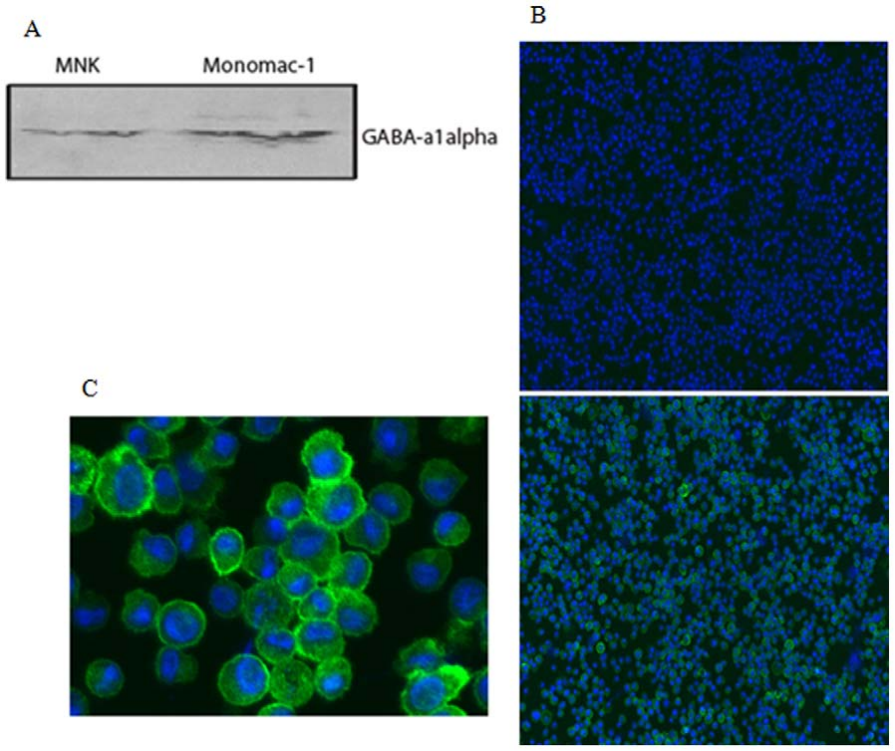
The presence of GABA_A_ receptors in monocytes and showing that GABA_A_ receptros are expressed on the cell surface of MonoMac1 cells. A) GABA_A_ 1 α receptors detected on MonoMac-1 cells and MNK cells by western blot after stimulation with securinine. B) MonoMac-1 cells were collected by Cytospin followed by fixation (75% Acetone/25% Ethanol). Cells were blocked in PBS containing 10% goat serum and 0.025% Tween 20. Slides were then stained with anti-GABA_A_ 1 α receptor mAb (20 ug/ml) for 30 min, washed with PBS containing 0.025% Tween 20, and then stained with AlexaFluor 488 goat anti-rabbit antibody. Slides were washed and treated with prolong Gold antifade reagent. DAPI was used to detected nuclei and the cells were analyzed by immunofluorescence microscopy. Green = GABA_A_ 1 α receptor and blue = DAPI. Top panel is DAPI alone. C) Higher magnification of the anti GABA_A_ 1 α stained Monomac I cells in the lower panel of B.

Figure 1A shows the results of a Western blot analysis of extracts of Monomac I cells, using antibodies against GABA_A_ receptors, compared to extracts of MNK cells (as a positive control) that demonstrates the presence of GABA_A_ receptors in both cell types. Figure 1B, bottom panel, shows a strong labeling signal in the Monomac I cells used in our studies, that was developed with a fluorescent antibody against GABA_A_ receptors, confirming the presence of GABA_A_ receptors in the MonomacI cells that indicates that most of the GABA_A_ receptors are located in the outer membrane of the cells.

### 2D Gel Analysis

**Figure 2:**
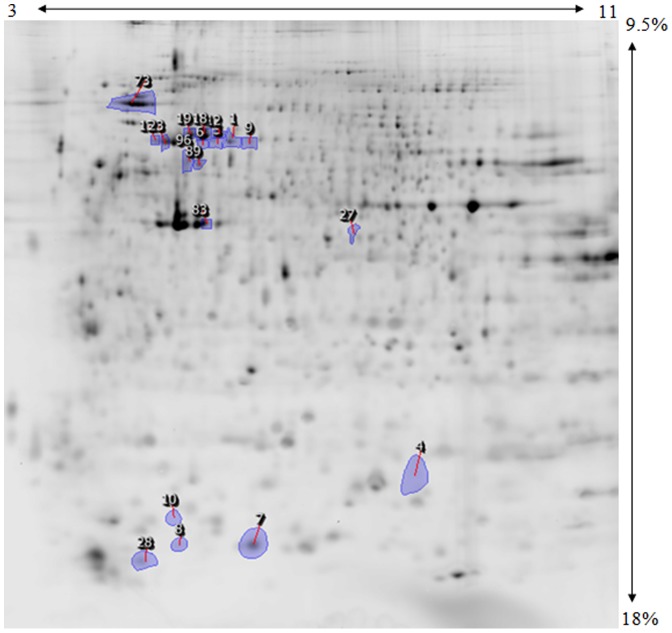
2D gel image of the soluble fraction from Securinine stimulated monocytes. Progenesis master image following image analysis and statistical analysis. Numbered spot areas indicate statistically significant differentially expressed spots, as determined by nested ANOVA analysis. The pI separation range (3-11NL) is the horizontal direction and the polyacrylamide gradient (9.5–18%) is the vertical direction. Spots 13 and and 23 are indicated by the arrows.

**Figure 3:**
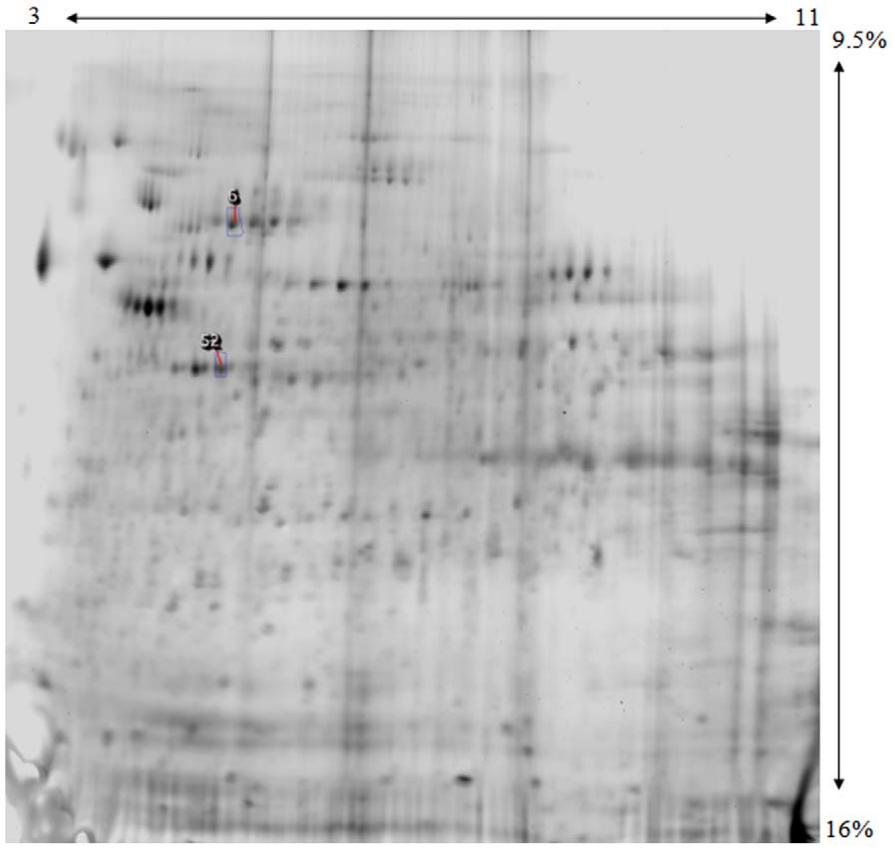
2D gel of the membrane fraction from Securinine stimulated monocytes. Progenesis master image, following image analysis and statistical analysis. Numbered spot areas indicate statistically significant differentially expressed spots, as determined by nested ANOVA analysis. The pI separation range (3-11NL) is in the horizontal direction and the polyacrylamide gradient (9.5–16%) is in the vertical direction.

Progenesis analysis of the soluble fraction of the cells+/−Securinine yielded 44 spots displaying a differential expression from −2.9 fold to +3.9 fold (p<0.05, observed power >0.8), as shown in Figure 2. The membrane fraction shows differential expression of 2 proteins as shown in Figure 3. The results were obtained from a total of 36 gel images from three biological replicates with three technical replicates in the ‘forward label’ condition (control ZGB, experimental ZBB) and three technical replicates in the ‘reverse label’ condition (control ZBB, experimental ZGB) for each biological replicate. The normalized spot volumes from Progenesis were exported for further analysis using the SPSS statistical software package (see Data S1 for normalized spot volumes).

Due to the combination of technical and biological replicates in our analysis, and the much lower sample variance in the technical replicates that could skew the statistical results, a method was needed to account appropriately for the technical and biological variations within the samples. Thus, we employed a general linear model, specifically a nested ANOVA analysis [21], as a suitable statistical method (as described in Shipman et al., submitted). Results from the nested ANOVA analysis revealed that Monomac I cells treated with Securinine displayed differential protein expression changes ranging from −2.9 to +3.9 fold, compared to untreated control cells. The nested ANOVA analysis found 22 differentially expressed protein spots as statistically significant in the soluble fraction, 20 (90%) of these were identified by mass spectrometry. The membrane fraction showed differential expression of 2 protein spots, both of which were identified.

### Bioinformatics

MS identifications and bioinformatics data for the differentially expressed proteins with statistically significant changes are shown in Tables 1 and 2. We concluded that half of the spots that were judged to be differentially regulated in the initial Progenesis analysis (when initially weighting technical and biological replicates equally) were judged to be differentially expressed in a statistically significant manner following the nested ANOVA analysis, underscoring the importance of properly accounting for technical and biological variation [21][Shipman, et al, submitted]. We found an average protein sequence coverage of ∼27% (range ∼16–46%) in the identified proteins over all of the bioinformatics tools employed. While the different bioinformatics tools gave substantially the same list of protein identifications, they did so with somewhat different lists of peptides for the identified proteins. This reflects differences in both the algorithms used and their treatment of posttranslational modifications. It should be noted that the global proteomic analysis used did not have the sensitivity to detect changes in the chemokine IL-8 that was shown to be elevated to about 2.5 ng/ml in Monomac I cells that were treated with 25 ug/ml Securinine or other selected protein changes that were detected by targeted Western analysis in human monocytes exposed to Securinine [1].

**Table 1:** Statistical summary and bioinformatics data for proteins that change expression in response to Securinine stimulation of monocytes.

Test condition	Spot #	Fold change	ID	p-value	power score
Sec sol	1	3.9	Hsp70 protein 1	0.0033	0.994
	3	3.4	Hsp70 protein 1	0.0028	0.997
	4	3	Fatty acid binding protein 5	0.0281	0.714
	5	2.9	L-plastin	0.0192	0.806
	7	−2.9	S100 Ca binding protein A4	0.0002	1
	8	−2.8	unidentified	0.0075	0.955
	9	2.7	Hsp70 protein 1	0.0107	0.913
	10	2.7	L-plastin	0.0068	0.963
	11	2.6	Thioredoxin	0.0077	0.951
	12	2.4	Hsp70 protein 8 isoform 1	0.0025	0.998
	13	−2.3	L-plastin	0.0002	1
	18	2.1	Hsp70 protein 8 isoform 1	0.0012	1
	19	2.1	Hsp70 protein 8 isoform 1	0.0220	0.775
	23	−2	L-plastin	0.0090	0.936
	27	1.9	Ser/cys proteinase inhibitor	0.0137	0.874
	28	−1.9	unidentified	0.0161	0.844
	73	1.5	Hsp90	0.0030	0.996
	83	1.5	Actin	0.0151	0.857
	89	1.5	Chaperonin/Hsp60	0.0171	0.831
	96	1.4	Inosine 5′ monophosphate dehydrogenase	0.0235	0.759
Sec mem	6	1.8	Hsp70	0.0009	1
	52	−1.3	Actin	0.0271	0.723

Statistical analysis of gel spot data. Normalized intensity values from each spot were analyzed using a mixed linear model in the SPSS v16.0 statistical package. The mixed linear model was a nested ANOVA analysis. The p-values and power scores as well as the fold changes observed in the gel spots are shown. “Sol” refers to the Securinine treated soluble fraction, “mem” refers to the Securinine treated membrane fraction.

**Table 2:** Bioinformatics data for each identified protein.

			Mascot			X!Hunter				P3			
Test condition	Spot #	ID	Score	Unique peptides	% coverage	Score	Unique peptides	% coverage	FDR (%)	Score	Unique peptides	% coverage	FDR (%)
Sec sol	1	Hsp70 protein 1	556	32	33.1	−137.3	14	23.7	0.31	−154.5	20	28.0	1.20
	3	Hsp70 protein 1	626	16	28.7	−74.4	9	17.2	0.42	−112.8	14	20.1	0.93
	4	Fatty acid binding protein 5	294	6	49.7	−27.9	6	43.7	0.38	−43.4	6	37.0	1.35%
	5	L-plastin	385	16	23.3	−76.5	11	19.9	0.83	−105.4	8	14.4	1.60
	7	S100 Ca binding protein A4	51	2	18.3	−8.8	2	18.8	0.41	not found	not found	not found	not found
	8	unidentified											
	9	Hsp70 protein 1	371	12	23.1	−94.9	9	16.3	0.34	−114.4	13	20.1	1.30%
	10	L-plastin	1405	32	43.6	−242.7	35	43.2	0.34	−238.7	34	43.9	1.14
	11	Thioredoxin				−15.5	2	22.9	0.24	−10.8	2	22.9	1.36
	12	Hsp70 protein 8 isoform 1	734	16	19.6	−152.0	14	30.2	0.41	−161.4	28	26.3	1.14
	13	L-plastin	1144	35	43.6	−147.8	17	28.2	0.45	−206.9	29	34.1	1.5
	18	Hsp70 protein 8 isoform 1	1403	34	36.4	−160.0	21	33.3	0.53	−188.0	30	31.1	1.15
	19	Hsp70 protein 8 isoform 1	825	15	19.1	−186.0	27	30.5	0.55	−211.8	36	29.3	1.07
	23	L-plastin	396	11	21.3	−89.3	9	18.8	0.47	−121.8	8	15.3	0.68
	27	SERPIN B1	313	9	26.9	−73.7	9	20.6	0.32	−71.1	9	21.4	1.27
	28	unidentified											
	73	Hsp90	1855	27	28.2	−247.4	40	28.0	0.18	−219.8	33	25.1	0.69
	83	Actin	281	10	22.2	−153.8	24	42.7	0.31	−120.7	16	38.1	1.23
	89	Chaperonin/Hsp60	722	16	33.4	−84.9	12	22.7	0.31	−122.1	9	21.5	1.08
	96	Inosine 5′ monophosphate dehydrogenase	439	10	22.4	−70.9	7	18.1	0.37	−44.4	5	12.5	1.45
Sec mem	6	Hsp70	583	19	28.9	−94.1	12	16.3	0.44	−122.8	19	23.4	1.54%
	52	β-actin	481	11	30.0	−125.8	16	36.8	0.57	−62.3	12	29.6	1.42%

Mascot scores were calculated by taking the sum of the Mascot peptide scores for all statistically significant peptides. All other protein scores are reported as provided by each bioinformatic tool. “Sol” refers to the Securinine treated soluble fraction, “mem” refers to the Securinine treated membrane fraction. Under the heading for each tool used, score refers to the score generated by the tool, unique peptides indicates the number of unique peptides found by the search, % coverage indicates the percentage of the identified protein's primary sequence that is covered by the data, and FDR refers to the False Discovery Rate.

It is possible that the major proteins identified in the 2D gel spots did not account for the major fraction of the fluorescence intensity differences that guided the selection of these spots for analysis. This possibility is unlikely for several reasons: the identified proteins gave strong MS signals that corresponded with the intensities of the protein levels in the spots of interest and the proteins identified were consistent with widespread evidence from the literature that rationalized their involvement in immune reactions. The proteins were soundly identified, since they had very high sequence homology scores that were much higher than any secondary candidates, the identified proteins showed high sequence coverage, and the identified proteins matched the experimental isoelectric points and molecular weights. See Data S2 for protein coverage maps.

### Systems biology modeling

**Figure 4:**
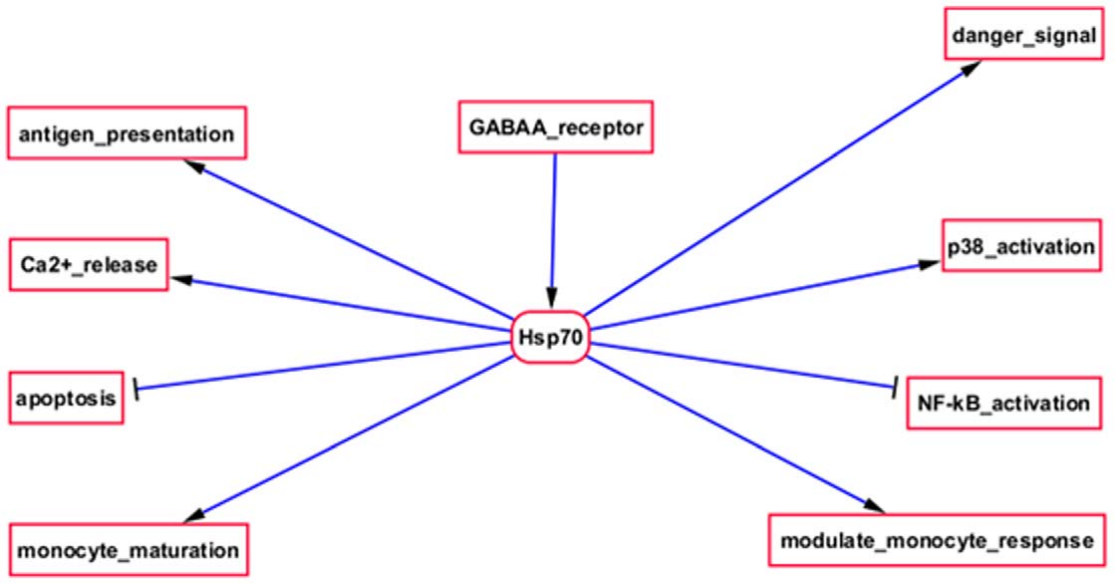
Schematic diagram of proposed functions of Hsp70 in Securinine stimulated monocytes, based on the literature. Proposed activities include increased monocyte maturation, inhibition of apoptosis, intracellular signaling (Ca^2+^, p38, NF-κB), and extracellular signaling (danger signal).

**Figure 5:**
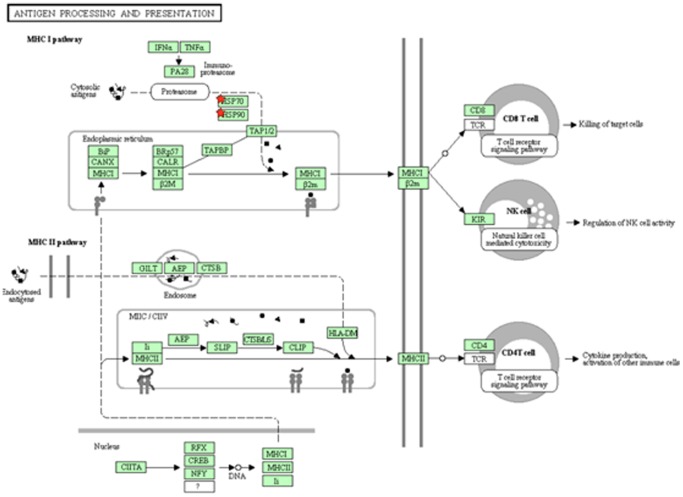
Schematic of antigen processing and presentation pathway for Hsp70 and Hsp90 generated by DAVID. DAVID utilized the KEGG pathways to diagram the role of Hsp70 and Hsp90 for antigen processing and presentation. Both Hsp70 and Hsp90 (red stars) are involved in MHC class I antigen processing. Hsp60 (not shown) is involved in MHC class II processing. The proteins and pathways proposed are in agreement with our manual search of the literature, which suggested that Hsps are involved in antigen presentation.

**Figure 6:**
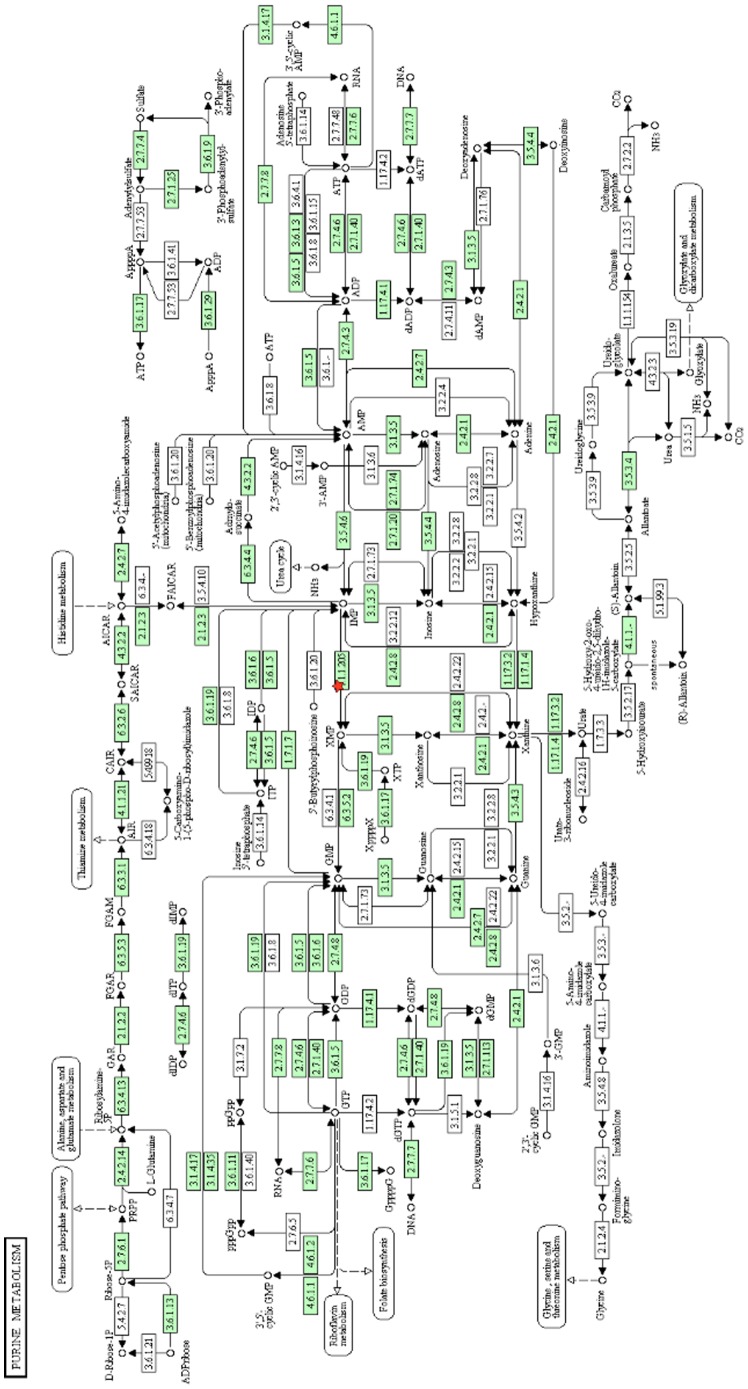
Schematic of metabolic pathways related to inosine 5′-monophosphate generated by DAVID. DAVID utilized KEGG pathways to depict metabolic pathways related to purine metabolism. Inosine 5′-monophosphate dehydrogenase (IMPDH, red star near the center of the figure) catalyzes the conversion of inosine 5′-monophosphate to xanthosine 5′-monophosphate which is thought to be the rate-limiting step for GMP and GTP production. Although the biological consequences of IMPDH are not detailed here, this diagram does offer an indicat-on of potential metabolites to monitor in targeted metabolomic experiments. The numbers in the boxes are enzyme (EC) numbers.

The proteins that were identified by the nested ANOVA analysis as differentially expressed in the Securinine stimulated monocytes, compared to controls, were used to guide a review of the literature. We developed a systems biology model of the mechanism of action of Securinine by graphing connections between interrelated proteins and their functions, as described in the literature. The resulting model is summarized in Figure S1 (displayed using Cytoscape [22]), which is presented in the supplementary material, since it was too large to fit as a readable text figure. Figure 4 shows details of a selected region of the overall model in Figure S1. The differentially expressed proteins were also analyzed using automated DAVID (Figures 5 and 6) [23], [24] and GOEAST (Figure S2) [25] systems biology analysis tools. See Table S1 for a list of accession numbers used to search GOEAST. The model, based on manual literature searches, was specifically focused on aspects of innate immunity, which our survey of the literature suggested were related to our target proteins, while the automated DAVID and GOEAST tools provided more details. Data from the automated pathway analyses generally supported the conclusions of the manually generated model, and also provided additional potential molecular connections (proteins and metabolites) that were not obtained by our survey of the literature, as discussed further below.

Briefly, the pathway analysis data from DAVID provides information from the Kyoto Encyclopedia of Genes and Genomes (KEGG) pathway analysis that points to relationships of fatty acid binding protein 5 (FABP5), inosine 5′-monophosphate dehydrogenase (IMPDH), actin, Hsp60, Hsp70, and Hsp90 proteins. Pathway data from GOEAST provides network diagrams of gene ontology (GO) terms for biological processes, molecular processes, and cellular localization. The information from all three network analysis approaches, manual, DAVID, and GOEAST was complimentary. DAVID provided a list of proteins interacting with the observed differentially expressed proteins. The automated systems were much more global in scope than the manual literature evaluations, but the automated results can include protein functions that are not likely to be relevant to the cells/system under investigation. For example, the GOEAST analysis indicated that actin is involved in axon formation. Axonogensis is not likely to be occurring in immune cells, and is thus extraneous to our analysis. However, cytoskeletal rearrangements of actin may well be relevant to the activated immune cells.

### Differentially expressed proteins

Representative gel images displaying differentially expressed protein spots are shown in Figures 2 and 3 and the differentially expressed proteins identified are shown in Table 2. L-plastin is present in several isoforms that are resolved on 2D gels. We find that some of the L-plastin isoforms are up-regulated and some are down-regulated, providing evidence for changes in the posttranslational modifications of this protein upon securinine stimulation. L-plastin is an actin bundling protein found in hematopoietic cells, and has been shown to activate integrins when phosphorylated at Ser5 [26]–[29]. Serpin B1, an intracellular member of the Serpin superfamily that functions in immunity [30], was upregulated in Securinine stimulated cells. Serpins act primarily as serine and cysteine protease inhibitors, frequently acting to prevent tissue damage and cell death [30]. Thioredoxin (Trx), is an intracellular redox enzyme, that we found to be upregulated in the soluble fraction of Securinine stimulated monocytes. Trx acts as a proton donor that catalyzes disulfide redox reactions [31], [32].

S100A4, a member of the S100 family of proteins, was observed to be downregulated in the soluble fraction of Securinine stimulated cells. A number of functions have been attributed to S100A4, including effects on cell motility, cytoskeletal rearrangements, signaling cascades, cancer progression, apoptosis, and cellular differentiation [33]. Hsps (60, 70, and 90), which have potential immunomodulatory functions [34]–[36], were found to be upregulated. In particular, a total of seven Hsp70 isoforms were observed to be upregulated, six in the soluble fraction and one in the membrane fraction. Differentially expressed proteins that appear to be of particular note in response to Securinine stimulation vs. control cells were fatty acid binding protein five (FABP5) and inosine-5′-monophosphate dehydrogenase (IMPDH). FABP5 is a lipid chaperone that sequesters and distributes lipids for regulating signaling and metabolism [37]–[39]. IMPDH catalyzes the NAD-dependant conversion of inosine 5′-monophosphate to xanthosine 5′ monophosphate, which is considered to be the rate limiting enzyme in GTP biosynthesis [40], [41], as diagrammed in Figure S1, and GTP is a key factor in many signaling pathways.

**Figure 7:**
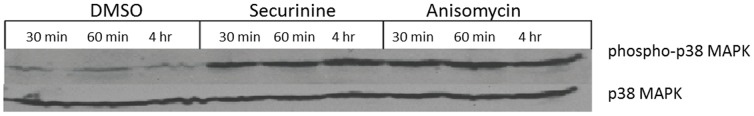
Securinine induces rapid phosphorylation of p38 MAPK in MonoMac-1 cells. MonoMac-1 cells were treated with DMSO, 50 uM securinine or 20 ug/mL anisomycin for the indicated times. Lysates were prepared and subjected to Western blot with anti-phospho-p38 MAPK (top panel) or anti-p38 MAPK (bottom panel). Blots were developed with ECL (GE Healthcare) and exposed to film for autoradiography. Anisomycin was used as a positive control.

Figure 7 demonstrates that treatment of Monomac I cells with Securinine induces a rapid phosphorylation of p38 MAPK. P38 is a common intracellular signaling molecule, that has many downstream effects. Treatment of Monomac I cells with DMSO induced no significant phosphorylation of p38. The intent of this experiment was to compare p38 phosphorylation status using the same cell treatment protocols as done in the proteome analysis: Securinine versus DMSO (buffer/carrier) treated cells. Results indicate that p38 was phosphorylated in the former, but not latter condition.

## Discussion

Analysis of changes in the 2D gel data before and after Securinine exposure has demonstrated a number of protein spots that are differentially expressed in a statistically significant manner. We have used the differentially expressed proteins in Table 2 to guide a search of the literature and have used the resulting information to develop an initial qualitative systems model to propose protein involvements in the response to Securinine stimulation. This approach has enabled us to propose signaling and metabolic pathways that may be involved in the monocytic response to Securinine exposure. Securinine is a GABA_A_ receptor antagonist and evidence has previously been presented that Securinine does not act through the Toll-like receptors in monocytes [1]. Application of Securinine has been shown to upregulate IL-8 [1]. GABA_A_ receptors are abundant in monocytes, as evidenced in Figure 1 [3], [4]. Recent proteomic analysis of lipopolysaccharide (LPS) stimulated TLRs in monocytes gave distinctly different proteome changes compared to those we find for Securinine exposure [6], [7], providing direct evidence that TLRs, and GABA_A_ receptors have different influences on the monocyte immune response.

Pereira et al., recently used 2D gels to examine the maturation of peripheral blood monocytes to dendritic cells, following exposure to GM-CSF and IL-4+/−LPS [42]. There was some relationship between the report of Pereira et al., and our results with regard to involvement of Hsp70, Hsp60, S100A4, and Thioredoxin, however, there were differences in the magnitude and/or even the direction of expression changes for all of the proteins that overlap in the two studies. Furthermore, there were quite different changes in the number and type of Hsp70 isoforms that were found to change [42]. Pereira et al., analyzed dendritic cells, whereas we and others [6], [7] tested monocytes, and the differences in monocyte maturation status may be responsible for the differences observed.

### Fatty acid binding protein 5

Fatty acid binding proteins (FABPs) are lipid chaperones that sequester and distribute lipids, resulting in regulation of signaling and enzymatic activities [43], that we find altered in our Securinine-stimulated samples. FABP5 is known to be upregulated in differentiated macrophages [38], and the upregulation of FABP5 in our Securinine stimulated samples may indicate that Securinine is inducing some level of monocyte maturation. FABP5 acts to coordinate and regulate metabolic and inflammatory activity in macrophages [37], [43], [44]. A reported increase in cholesterol esterfication in response to the upregulation of FABP5 [65] suggests that the amount of cholesterol available for lipid rafts may decrease in the presence of Securinine.

Lipid rafts are discrete regions of the membrane that are highly enriched in cholesterol and are believed to serve as important sites for vesicle trafficking, protein docking, signaling and other functions [45]–[47]. Increased cholesterol esterification will tend to reduce the amount of free cholesterol available in the cell and could alter the cholesterol content of lipid rafts, potentially affecting protein binding to lipid rafts [48]. The manually generated model suggests that lipid composition and several metabolites are being altered within the cell in response to Securinine exposure, and a direct measurement of the levels of these metabolite compounds may be beneficial in future experiments.

An increase in FABP5 expression has been shown to cause a reduction in short chain fatty acids and an increase in longer chain fatty acids [37], [49], suggesting that lipid metabolism, and possibly energy balance, may be altered by Securinine stimulation. FABP5 expression is correlated with some decrease in insulin sensitivity and increases in inflammation [37], [43], [44], and higher levels of FABP5 lead to a decrease in glucose uptake, even when insulin levels remain constant [37], [43], [44]. Metabolic pathways that lead to energy mobilization (e.g. glycogen degradation), that can be activated by hormonal stimulation, typically by triggering the production of cAMP, which activates intracellular signaling cascades. Insulin causes cAMP to be degraded into AMP, short-circuiting cAMP-mediated signaling cascades. By inhibiting insulin sensitivity, increased FABP5 will tend to reduce insulin inhibition of hormonally regulated metabolic pathways. Changes in FABP5 may thus have significant implications for insulin sensitive metabolic and signaling pathways in Securinine stimulated cells. FABP5 may thus be involved in regulation of lipid metabolism, influencing lipid raft composition, inflammation, and hormonal regulation of monocytes, as indicated in the diagram of our model in Figure S1, and suggests that FABP5 could be an important regulator of cellular metabolism in activated monocytes.

### Heat shock proteins

In view of the suggestion that changes in lipid raft composition might be associated with Securinine exposure, evidence from the literature for involvement of Hsp 70 and 90 with lipid rafts is intriguing [50], [51]. Binding of Hsp70 to macrophage lipid rafts was reported to increase phagocytosis in a dose-dependent manner [51], and there have also been indications that lysosomal lipid rafts may be involved in nonclassical Hsp70 secretion pathways [35], [52], as well as Hsp70 localization and recognition at the cell surface [52]. Therefore, characterization of changes in monocyte lipid rafts in response to Securinine stimulation could prove informative.

Figure 4 and Figure S1 diagram functions that have been proposed for Hsp60 and 70 with regard to their involvement in antigen presentation. Hsp60 can insert into the membrane as part of the antigen presentation machinery of macrophages, where it can stimulate a Th1-type T-cell adaptive response in response to infection [53], [54]. Hsp70 interaction with macrophage lipid rafts has been reported to facilitate antigen presentation to CD4^+^ T-cells in an MHC-II-dependent manner [51], [55], as well as activation of cognate T cells in an MHC-I-dependent manner [56]. Reports have also indicated that Hsp90 is involved in antigen presentation. Binder et al., [57], [58], demonstrated that peptides chaperoned by Hsp70 and Hsp90 were presented in an MHC class I-dependant manner much more efficiently than free cytosolic peptides. It was also shown that loss of Hsp90 alone did not inhibit MHC I presentation, suggesting redundancy with Hsp70 in this pathway [34], [59].

Hsp90 has been shown to have a number of client proteins, which include a large number of transcription factors, kinases, and cell cycle regulators leading to the hypothesis that Hsp90 is a chaperone regulating key proteins in signaling pathways [60], [61]. Hsp90 is believed to maintain client proteins in an unfolded (and inactive), but activatable state, which allows rapid mobilization of responses of the client's proteins to various signals [60], [61]. When signaled to do so, the unfolded, inactive client may be quickly released from Hsp90 to fold into its active state [60], [61]. Hsp90 also interacts with Hsp70, which is believed to help deliver client proteins to Hsp90 [62].

Understanding of the effects of Securinine on Hsp90 binding of client proteins might well provide a deeper understanding of the effects of Securinine on intracellular signaling pathways. Hsp90 has been shown to be involved in activation of macrophages by enabling induction of NF-κB translocation to the nucleus [63], as well as activation of p38, JNK, and ERK pathways [64], suggesting that Hsp90 has an important role in innate immune function in general, as diagrammed in Figure S1. Signaling pathway proteins that are below the detection limit of the fluorescent dyes, as applied in these experiments, perhaps could be found bound to Hsp90, using immunoprecipitation experiments.

Hsp60 and 70 appear to influence the inflammatory state of the monocytes. It has been reported that Hsp70 and Hsp60 may be stimulating the production and export of inflammatory signals, such as TNF-α and other cytokines [34], [51], [58], thus playing a role in modulating the inflammatory response. Cytokine modulation by Hsp60 and Hsp70 was shown to involve p38 signaling pathways, in response to endotoxin [65]. Previously reported results have indicated that Hsp60 and Hsp70 can also be secreted and act as cytokines, attracting nearby immune cells, and potently activating both macrophage maturation and the immune system [34], [51], [58]. Export under immune stimulated conditions has led to proposals that Hsps 60 and 70 can act as ‘danger signals’ in the immune system [66], [67].

### Inosine 5′-monophosphate dehydrogenase (IMPDH)

Guanine nucleotides produced by IMPDH are required for both NO production and TNFα production in macrophages [68], and the upregulation of IMPDH in the Securinine stimulated monocytes would be consistent with upregulation of NO and TNFα. It has been reported that IMPDH is a factor in stimulation of the p38 pathway [69], [70]. IMPDH is not the only component that affects p38 activation, but could be involved in the activation of p38 observed in our Securinine stimulated samples, as shown in Figure 7. It has been shown that activation of isolated peripheral blood monocytes, using phorbol-12-myristate-13-acetate and either ionomycin or pokeweed mitogen, causes an upregulation of IMPDH mRNA 24 hours after stimulation [40]. It was also suggested that induction of IMPDH in monocytes plays a role in cell proliferation [40], [71], as diagrammed in Figure S1. It was demonstrated that IMPDH expression and activity were upregulated upon T cell activation [71], and upregulation of IMPDH may be a general response to immune activation. We suggest that increased expression of IMPDH is influencing the inflammatory response, as well as possibly regulating p38 activation and monocyte differentiation in the securinine stimulated cells. Quantifying the activity of IMPDH in our system [72], as well as the level of GTP could be informative as to the potential impact of IMPDH on the biology of Securinine stimulated monocytes.

### L-plastin

L-plastin was found to be downregulated in two spots (spots 13 and 23), and upregulated in one spot (spot 6). The pattern observed on the gel suggests that spots 13 and 23 have been dephosphorylated. Sustained integrin signaling by increased L-plastin is likely to be part of the Securinine-induced stimulation of p38 signaling, as diagrammed in Figure S1. The observation by Chen et al., that L-plastin^−/−^ mice were defective in killing *Staphylococcus aureus*, was thought to stem from a lack of β2-integrin signaling [26]. L-plastin is not required for activation of β2-integrin, but L-plastin is required to help stabilize the signal from β2-integrin that gives rise to the bactericidal respiratory burst of superoxide, which results from activation of β2-integrin. L-plastin can regulate cytoskeletal rearrangements to create conditions favorable to formation of both an integrin signaling complex [26] as well as for formation of the NADPH oxidase complex, which are central to the antimicrobial respiratory burst [73].

Interestingly, L-plastin contains two EF-hand Ca^2+^ binding domains, the same type of Ca^2+^ binding motif found in the Ca^2+^ binding protein S100A4 [74], [75]. This suggests the possibility that L- plastin may be involved in regulating intracellular signaling in response to Ca^2+^. Jones et al., suggest that PI_3_K is directly involved in phosphorylation of L-plastin [27]. This may be particularly relevant considering that PI_3_K also appears to be involved in regulating Rab7 activities in phagolysosome formation, an important event for resistance to obligate intracellular pathogen infection of monocytes (Shipman, et al., submitted).

L-plastin is unique among the plastin family in that it is regulated by phosphorylation at Ser5 and Ser7 [73]. Phosphorylation of proteins causes a distinct acidic shift in the position of the phosphorylated protein isoforms on 2D gels, however other posttranslational modifications also shift protein positions on 2D gels. Once activated, integrins can stimulate the p38 inflammation pathway [76]. As shown in Figure 7, we have observed that Securinine stimulates p38 activation. We were not able to distinguish phosphorylation site occupation in these studies with the methods used, and the role of protein modifications could serve as targets for future studies.

### Thioredoxin (Trx)

Trx is upregulated in response to Securinine stimulation of monocytes. The principal function of Trx is to protect against oxidative stress, by reducing oxidized disulfides in enzyme active sites [32], [77], [78]. The active site of Trx must be kept in a reduced state to maintain enzyme activity and this reduction is achieved by an NADPH-dependant Trx reductase enzyme [31], [79]. Trx acts primarily intracellularly but is also secreted to act extracellularly through a nonclassical export pathway [31], [32]. Secreted Trx possesses chemokine properties and serves to attract monocytes, T cells, and polymorphonuclear leukocytes [31], [32]. Trx involvement with signaling in the cytosol includes interaction with Ask1, an apoptosis-inducing kinase [80]. In the reduced form, Trx binds to and inhibits Ask1 activity [80]. When Trx is in the oxidized form, it dissociates from Ask1, thus freeing Ask1 to influence p38, JNK, and contribute to TNFα-induced apoptosis [80]. Changes in Trx activity have implications for a number of redox-dependent cellular mechanisms, including signal transduction, inflammation, and apoptosis [31], [32], [78], [81], as diagrammed in Figure S1.

### S100A4

Downregulation of S100A4 by Securinine stimulation suggests that cell motility could be reduced and that there may be alterations to the cytoskeleton [82]. It has been reported that Hsp70 can stimulate S100A4 secretion [83]. Given the elevated levels of Hsp70 in in both the soluble and membrane fractions of the Securinine stimulated cells, it seems likely that we are observing downregulation of S100A4 in the soluble fraction, due to an increase in secretion. If secretion of S100A4 is occurring, it could be participating in the activation of p38. It could be beneficial to determine if there are changes in S100A4 secretion and other secreted proteins by measuring the secretome of Securinine stimulated monocytes.

It has been observed that S100A4 is more highly expressed in cells with high motility [84]. S100 proteins have no known enzymatic function, but exert their influence by binding to and modifying the activity of other proteins, following activation by Ca^2+^ binding [84], [85]. S100A4 has been implicated in binding to target proteins such as p53 and inhibiting their phosphorylation [86]. Various other binding partners of S100A4 have been identified, including nonmuscle myosin heavy chain IIA, liprin β1, RAGE, annexins, and actin [87]–[90]. These binding partners support various functionalities related to the cytoskeleton, including motility, and inflammation. S100A4 has also been shown to be secreted and to act extracellularly [33], [91]. Among the activities reported for extracellular S100A4 is activation of NF-κB, that is mediated by membrane receptors, by inducing phosphorylation of IκBα. Phosphorylation of IκBα triggers its degradation and subsequent release of NF-κB, as well as activation of the p38, ERK1/2, and JNK pathways [33].

### SERPINB1

We found that Serpin B1 is upregulated by Securinine stimulation. Transcription of Serpin B1 is known to be regulated by NF-kB [92], [93], suggesting that the some of the same proteins that regulate inflammation also regulate Serpin B1. Figure 7 shows that Securinine quickly induces phosphorylation of p38, suggesting that Securinine-induced phosphorylation of p38 may initiate the upregulation of Serpin B1. It also seems reasonable that Serpin B1 is acting in a protective capacity, since upregulation of Serpin B1 has a positive effect on bacterial clearance [94]. Upregulation of Serpin B1 would allow for a stronger inflammatory response following Securinine exposure.

SerpinB1, also referred to as Monocyte Neutrophil Elastase Inhibitor (MNEI), inhibits elastase, cathepsin G and proteinase 3 proteases [30], [95], [96]. Serpins are involved in cell migration, phagocytosis, and cell killing of bacteria and viruses [30], [94], [97]. SerpinB1 is an efficient suicide inhibitor, acting at a stoichiometry of 1∶1 with target proteases [30], [93], [95]. Knockout of Serpin B1 expression causes reduced effectiveness of the immune response [94]. Serpin B1 seems to act as an inflammation modulator, by limiting the damage done to host cell proteins during inflammation by preventing degradation of immune active proteins [98], and thereby facilitating a stronger immune response.

## Conclusions

Our results on differential protein expression combined with information from the literature suggest that GABA_A_ receptors and TLRs have different regulatory functions in the monocyte immune responses. Given the probable inhibitory role of GABA_A_ receptors on the innate immune response [5], our data suggests the hypothesis that exposure of monocytes to Securinine is removing an inhibitor to monocyte immune function, thus stimulating the innate immune system. Removal of inhibition of monocyte activation is proposed to be causing modulation of antigen presentation machinery, changing the cytokine/chemokine profiles and metabolite levels within the cells, in addition to triggering maturation of the monocytes into macrophages or dendritic cells.

We were not able to detect changes in the cytokine profiles in response to Securinine treatment on 2D gels, but they have been previously reported [1]. Securinine has been shown to activate IL-8 [1], and this potent cytokine may play a role in the monocyte activation described above with GABA_A_ inhibition likely amplifying the effects of cytokine stimulation. Cytokines are secreted signaling molecules that occur at relatively low abundance, and thus would have been most prevalent in the growth media, rather than in the soluble fraction, where more abundant proteins presumably obscured changes in cytokine abundance. We did not extract the growth media or otherwise examine the secreted proteins in these experiments, and this may also be a reason that we did not observe changes in cytokine abundance.

As GABA is an inhibitory neurotransmitter and Securinine is quite selective for GABA_A_ receptors [1], [2], we propose that GABA-stimulated monocytes act to dampen the immune response, providing a self-limiting, inhibitory regulation system in a manner similar to that reported for T cells [5]. It is most likely that the GABA is coming from the bloodstream, as GABA is reported to be present in plasma at 100 nM, a concentration similar to that found in nerve synapses [5]. A recent study by Bjurstöm et al., reported that GABA receptors on T cells inhibited proliferation at physiologically relevant GABA concentrations [5]. A central hypothesis derived from the present work is that application of Securinine prevents GABA_A_ receptor-mediated inhibition of the monocyte immune response, increasing the effectiveness of the monocyte response and thereby increasing the monocytic cell killing of *C. burnetii* [1] and potentially other pathogens. Additonal work will be required to more fully characterize the proteins and protein isoforms involved in the response to Securinine, to confirm the involvement of the proteins identified and to expand and test the systems biology model.

Securinine represents a potential new class of innate immune stimulators. Securinine has been used clinically for treatment of neurological disorders, including multiple sclerosis [99]. The botanical source of Securnine, *Securinega suffruticosa*, is one of the 50 fundamental herbs in Chinese medicine and has long been recognized to have potent biological properties [99]. Further evaluation of Securinine or Securinine analogs as treatment options either alone, or in conjunction with existing treatments (e.g. antibiotics), could help to improve the prognosis for those affected by *C. burnetii* and other infectious organisms, particularly obligate intracellular pathogens. Our work is consistent with the possibility that statins may also be beneficial against *C. burnetii* infection (and other obligate intracellular pathogens) [48], [100], [101], possibly in combination with Securinine. Because adjuvants do not rely on specific antigens to stimulate immune responses, and can work much more rapidly than vaccines, it is possible that Securinine or similar compounds could be deployed rapidly to the site of an infectious disease outbreak to help maintain the effectiveness of emergency personnel and to help limit the spread of disease.

## Supporting Information

Data S1 **Normalized spot volumes.** The contents of this file contain the normalized spot volumes exported from Progenesis. Securinine soluble fraction was analyzed using Progenesis version PG240. Securinine membrane fraction was exported from Progenesis version 3. Each normalized spot volume represents a measurement made from an individual gel. In Progenesis, each spot was required to pass an initial evaluation with statistical cutoffs of p<0.05 and power scores ≥0.8. Data was exported to Excel and then input into SPSS v.16 for final statistical analysis using a nested ANOVA approach (see text of paper for details). In the final analysis by SPSS, a spot was deemed as passing analysis if it met cutoffs of P<0.05, power score >0.7. Whether a spot passed or failed SPSS analysis is indicated by Pass or Fail next to each spot number.
(XLS)

Data S2 **Protein coverage maps.** Coverage maps of proteins determined by bioinformatics analysis. The primary sequence is listed for each map. Sections highlighted in red indicate the regions of the protein where peptides were identified. The bioinformatics tool used is indicated along with the protein identity and spot number. In order to be considered an identity, a peptide required at least 50% sequence coverage.
(PDF)

Figure S1 **Systems biology network model proposed for Monomac I cellular response to stimulation by Securinine.** Graph of the network model generated by the manual curation of the literature. See text for details of this model.
(PDF)

Figure S2 **GOEAST model showing the molecular functions of the differentially expressed proteins from Securinine stimulated Monomac I cells.** In this image, a sample of the data from GOEAST searches are displayed. The data displayed shows a search of GO terms related to molecular functions for the differentially expressed proteins in the present investigation. A) complete graph from GOEAST showing various connections. Yellow squares indicate that at least one accession number from supplemental Table 1 was identified for that GO term. White squares indicate that no accession numbers were found. Red arrows go from one detected accession number to another (can be the same term for more than one function), while the black arrows extend from a GO term which no accession numbers were associated with.
(PDF)

Table S1 **List of accession numbers used to search GOEAST.**
(XLS)

Methods S1 **Additional details of methodology, with citations** **[102]**–**[106]** **.**
(DOC)
