# Supplementary material for: Proteomic and Systems Biology Analysis of Monocytes Exposed to Securinine, a GABAA Receptor Antagonist and Immune Adjuvant
Source: PLoS One. 2012 Sep 13;7(9):e41278. doi: 10.1371/journal.pone.0041278 (PMC3441550; doi:10.1371/journal.pone.0041278)
Supplement: Data S2 — Protein coverage maps. Coverage maps of proteins determined by bioinformatics analysis. The primary sequence is listed for each map. Sections highlighted in red indicate the regions of the protein where peptides were identified. The bioinformatics tool used is indicated along with the protein identity and spot number. In order to be considered an identity, a peptide required at least 50% sequence coverage. (PDF) [file pone.0041278.s002.pdf]

Securinine soluble fraction/Spot 1/Hsp70/Mascot
LRSCCDSPLPFSRVTPVVPRLPRANLCGCRHRRVEFPASGRTELFSRIQCSVSSPQSQSRADREQGTGMAKAAAIGIDL
GTTYSCVGVFQHGKVEIIANDQGNRTTPSYVAFTDTERLIGDAAKNQVALNPQNTVFDAKRLIGRKFGDPVVQSDMK
HWPFQVINDGDKPKVQVSYKGDTKAFYPEEISSMVLTKMKEIAEAYLGYPVTNAVITVPAYFNDSQRQATKDAGVIA
GLNVLRIINEPTAAAIAYGLDRTGKGERNVLIFDLGGGTFDVSILTIDDGIFEVKATAGDTHLGGEDFDNRLVNHFVEEF
KRKHKKDISQNKRAVRRLRTACERAKRTLSSSTQASLEIDSLFEGIDFYTSITRARFEELCSDLFRSTLEPVEKALRDAKLDK
AQIHDLVLVGGSTRIPKVQKLLQDFFNGRDLNKSINPDEAVAYGAAVQAAILMGDKSENVQDLLLLDVAPLSLGLETA
GGVMTALIKRNSTIPTKQTQIFTTYSDNQPGVLIQVYEGERAMTKDNNLLGRFELSGIPPAPRGVPQIEVTFDIDANGIL
NVTATDKSTGKANKITITNDKGRLSKEEIESMVQEAEKYKAEDEVQRERVSAKNALESYAFNMKSAVEDEGLKGKISEA
DKKKVLDKCQEVISWLDANTLAEKDEFEHKRKELEQVCNPIISGLYQGAGGPGPGGFGAQGPKGGSGSGPTIEEVD

Securinine soluble fraction/Spot 1/Hsp70/X!Hunter
MAKAAAIGIDLGTTYSCVGVFQHGKVEIIANDQGNRTTPSYVAFTDTERLIGDAAKNQVALNPQNTVFDAKRLIGRKFG
DPVVQSDMKHWPFQVINDGDKPKVQVSYKGETKAFYPEEISSMVLTKMKEIAEAYLGYPVTNAVITVPAYFNDSQRQ
ATKDAGVIAGLNVLRIINEPTAAAIAYGLDRTGKGERNVLIFDLGGGTFDVSILTIDDGIFEVKATAGDTHLGGEDFDNRL
VNHFVEEFKRKHKKDISQNKRAVRRLRTACERAKRTLSSSTQASLEIDSLFEGIDFYTSITRARFEELCSDLFRSTLEPVEKAL
RDAKLDKAQIHDLVLVGGSTRIPKVQKLLQDFFNGRDLNKSINPDEAVAYGAAVQAAILMGDKSENVQDLLLLDVAPLS
LGLETAGGVMTALIKRNSTIPTKQTQIFTTYSDNQPGVLIQVYEGERAMTKDNNLLGRFELSGIPPAPRGVPQIEVTFDID
ANGILNVTATDKSTGKANKITITNDKGRLSKEEIERMVQEAEKYKAEDEVQRERVSAKNALESYAFNMKSAVEDEGLKG
KISEADKKKVLDKCQEVISWLDANTLAEKDEFEHKRKELEQVCNPIISGLYQGAGGPGPGGFGAQGPKGGSGSGPTIEE
VD

Securinine soluble fraction/Spot 1/Hsp70/X!Tandem P3
MAKAAAIGIDLGTTYSCVGVFQHGKVEIIANDQGNRTTPSYVAFTDTERLIGDAAKNQVALNPQNTVFDAKRLIGRKFG
DPVVQSDMKHWPFQVINDSQRQATKDAGVIAGLNVLRIINEPTAAAIAYGLDRTGKGERNVLIFDLGGGTFDVSILTID
DGIFEVKATAGDTHLGGEDFDNRLVNHFVEEFKRKHKKDISQNKRAVRRLRTACERAKRTLSSSTQASLEIDSLFEGIDFY
TSITRARFEELCSDLFRSTLEPVEKALRDAKLDKAQIHDLVLVGGSTRIPKVQKLLQDFFDGRDLNKSINPDEAVAYGAAV
QAAILMGDKSENVQDLLLLDVAPLSLGLETAGGVMTALIKRNSTIPTKQTQIFTTYSDNQPGVLIQVYEGERAMTKDNN
LLGRFELSGIPPAPRGVPQIEVTFDIDANGILNVTATDKSTGNANKITITNDKGRLSKEEIERMVQEAEKYKAEDEVQRER
VSAKNALESYAFNMKSAVEDEGLKGKISEADKKKVLDKCQEVISWLDANTLAEKDEFEHKRKELEQVCNPIISGLYQGAG
GPGPGGFGAQGPKGGSGSGPTIEEVD

Securinine soluble fraction/Spot 3/Hsp70/Mascot
LRSCCDSPLPFSRVTPVVPRLPRANLCGCRHRRVEFPASGRTELFSRIQCSVSSPQSQSRADREQGTGMAKAAAIGIDL
GTTYSCVGVFQHGKVEIIANDQGNRTTPSYVAFTDTERLIGDAAKNQVALNPQNTVFDAKRLIGRKFGDPVVQSDMK
HWPFQVINDGDKPKVQVSYKGDTKAFYPEEISSMVLTKMKEIAEAYLGYPVTNAVITVPAYFNDSQRQATKDAGVIA
GLNVLRIINEPTAAAIAYGLDRTGKGERNVLIFDLGGGTFDVSILTIDDGIFEVKATAGDTHLGGEDFDNRLVNHFVEEF
KRKHKKDISQNKRAVRRLRTACERAKRTLSSSTQASLEIDSLFEGIDFYTSITRARFEELCSDLFRSTLEPVEKALRDAKLDK
AQIHDLVLVGGSTRIPKVQKLLQDFFNGRDLNKSINPDEAVAYGAAVQAAILMGDKSENVQDLLLLDVAPLSLGLETA
GGVMTALIKRNSTIPTKQTQIFTTYSDNQPGVLIQVYEGERAMTKDNNLLGRFELSGIPPAPRGVPQIEVTFDIDANGIL
NVTATDKSTGKANKITITNDKGRLSKEEIESMVQEAEKYKAEDEVQRERVSAKNALESYAFNMKSAVEDEGLKGKISEA
DKKKVLDKCQEVISWLDANTLAEKDEFEHKRKELEQVCNPIISGLYQGAGGPGPGGFGAQGPKGGSGSGPTIEEVD

Securinine soluble fraction/Spot 3/Hsp70/X!Hunter
MAKAAAIGIDLGTTYSCVGVFQHGKVEIIANDQGNRTTPSYVAFTDTERLIGDAAKNQVALNPQNTVFDAKRLIGRKFG
DPVVQSDMKHWPFQVINDGDKPKVQVSYKGETKAFYPEEISSMVLTKMKEIAEAYLGYPVTNAVITVPAYFNDSQRQ
ATKDAGVIAGLNVLRIINEPTAAAIAYGLDRTGKGERNVLIFDLGGGTFDVSILTIDDGIFEVKATAGDTHLGGEDFDNRL
VNHFVEEFKRKHKKDISQNKRAVRRLRTACERAKRTLSSSTQASLEIDSLFEGIDFYTSITRARFEELCSDLFRSTLEPVEKAL
RDAKLDKAQIHDLVLVGGSTRIPKVQKLLQDFFNGRDLNKSINPDEAVAYGAAVQAAILMGDKSENVQDLLLLDVAPLS
LGLETAGGVMTALIKRNSTIPTKQTQIFTTYSDNQPGVLIQVYEGERAMTKDNNLLGRFELSGIPPAPRGVPQIEVTFDID
ANGILNVTATDKSTGKANKITITNDKGRLSKEEIERMVQEAEKYKAEDEVQRERVSAKNALESYAFNMKSAVEDEGLKG
KISEADKKKVLDKCQEVISWLDANTLAEKDEFEHKRKELEQVCNPIISGLYQGAGGPGPGGFGAQGPKGGSGSGPTIEE
VD

Securinine soluble fraction/Spot 3/Hsp70/X!Tandem P3
MAKAAAIGIDLGTTYSCVGVFQHGKVEIIANDQGNRTTPSYVAFTDTERLIGDAAKNQVALNPQNTVFDAKRLIGRKFG
DPVVQSDMKHWPFQVINDGDKPKVQVSYKGETKAFYPEEISSMVLTKMKEIAEAYLGYPVTNAVITVPAYFNDSQRQ
ATKDAGVIAGLNVLRIINEPTAAAIAYGLDRTGKGERNVLIFDLGGGTFDVSILTIDDGIFEVKATAGDTHLGGEDFDNRL
VNHFVEEFKRKHKKDISQNKRAVRRLRTACERAKRTLSSSTQASLEIDSLFEGIDFYTSITRARFEELCSDLFRSTLEPVEKAL
RDAKLDKAQIHDLVLVGGSTRIPKVQKLLQDFFNGRDLNKSINPDEAVAYGAAVQAAILMGDKSENVQDLLLLDVAPLS
LGLETAGGVMTALIKRNSTIPTKQTQIFTTYSDNQPGVLIQVYEGERAMTKDNNLLGRFELSGIPPAPRGVPQIEVTFDID
ANGILNVTATDKSTGKANKITITNDKGRLSKEEIERMVQEAEKYKAEDEVQRERVSAKNALESYAFNMKSAVEDEGLKG
KISEADKKKVLDKCQEVISWLDANTLAEKDEFEHKRKELEQVCNPIISGLYQGAGGPGPGGFGAQGPKGGSGSGPTIEE
VD

Securinine soluble fraction/Spot 4/Fatty acid binding protein 5/Mascot
MATVQQLEGRWRLVDSKGFDEYMKELGVGIALRKMGAMAKPDCIITCDGKNLTIKTESTLKTTQFSCTLGEKFEETTA
DGRKTQTVCNFTDGALVQHQEWDGKESTITRKLKDGKLVVECVMNNVTCTRIYEKVE

Securinine soluble fraction/Spot 4/Fatty acid binding protein 5/X!Hunter
MATVQQLEGRWRLVDSKGFDEYMKELGVGIALRKMGAMAKPDCIITCDGKNLTIKTESTLKTTQFSCTLGEKFEETTAD
GRKTQTVCNFTDGALVQHQEWDGKESTITRKLKDGKLVVECVMNNVTCTRIYEKVE

Securinine soluble fraction/Spot 4/Fatty acid binding protein 5/X!Tandem P3
MATVQQLEGRWRLVDSKGFDEYMKELGVGIALRKMGAMAKPDCIITCDGKNLTIKTESTLKTTQFSCTLGEKFEETTAD
GRKTQTVCNFTDGALVQHQEWDGKESTITRKLKDGKLVVECVMNNVTCTRIYEKVE

Securinine soluble fraction/Spot 5/L-plastin/Mascot
MARGSVSDEEMMELREAFAKVDTDGNGYISFNELNDLFKAACLPLPGYRVREITENLMATGDLDQDGRISFDEFIKIF
HGLKSTDVAKTFRKAINKKEGICAIGGTSEQSSVGTQHSYSEEEKYAFVNWINKALENDPDCRHVIPMNPNTNDLFNA
VGDGIVLCKMINLSVPDTIDERTINKKKLTPFTIQENLNLALNSASAIGCHVVNIGAEDLKEGKPYLVLGLLWQVIKIGLF
ADIELSRNEALIALLREGESLEDLMKLSPEELLLRWANYHLENAGCNKIGNFSTDIKDSKAYYHLLEQVAPKGDEEGVPA
VVIDMSGLREKDDIQRAECMLQQAERLGCRQFVTATDVVRGNPKLNLAFIANLFNRYPALHKPENQDIDWGALEGET
REERTFRNWMNSLGVNPRVNHLYSDLSDALVIFQLYEKIKVPVDWNRVNKPPYPKLGGNMKKLENCNYAVELGKNQ
AKFSLVGIGGQDLNEGNRTLTLALIWQLMRRYTLNILEEIGGGQKVNDDIIVNWVNETLREAEKSSSISSFKDPKISTSLP
VLDLIDAIQPGSINYDLLKTENLNDDEKLNNAKYAISMARKIGARVYALPEDLVEVNPKMVMTVFACLMGKGMKRV

Securinine soluble fraction/Spot 5/L-plastin/X!Hunter
MARGSVSDEEMMELREAFAKVDTDGNGYISFNELNDLFKAACLPLPGYRVREITENLMATGDLDQDGRISFDEFIKIFH
GLKSTDVAKTFRKAINKKEGICAIGGTSEQSSVGTQHSYSEEEKYAFVNWINKALENDPDCRHVIPMNPNTNDLFNAVG
DGIVLCKMINLSVPDTIDERTINKKKLTPFTIQENLNLALNSASAIGCHVVNIGAEDLKEGKPYLVLGLLWQVIKIGLFADIE
LSRNEALIALLREGESLEDLMKLSPEELLLRWANYHLENAGCNKIGNFSTDIKDSKAYYHLLEQVAPKGDEEGVPAVVID
MSGLREKDDIQRAECMLQQAERLGCRQFVTATDVVRGNPKLNLAFIANLFNRYPALHKPENQDIDWGALEGETREER
TFRNWMNSLGVNPRVNHLYSDLSDALVIFQLYEKIKVPVDWNRVNKPPYPKLGGNMKKLENCNYAVELGKNQAKFSL
VGIGGQDLNEGNRTLTLALIWQLMRRYTLNILEEIGGGQKVNDDIIVNWVNETLREAEKSSSISSFKDPKISTSLPVLDLID
AIQPGSINYDLLKTENLNDDEKLNNAKYAISMARKIGARVYALPEDLVEVNPKMVMTVFACLMGKGMKRV

Securinine soluble fraction/Spot 5/L-plastin/X!Tandem P3
MARGSVSDEEMMELREAFAKVDTDGNGYISFNELNDLFKAACLPLPGYRVREITENLMATGDLDQDGRISFDEFIKIFH
GLKSTDVAKTFRKAINKKEGICAIGGTSEQSSVGTQHSYSEEEKYAFVNWINKALENDPDCRHVIPMNPNTNDLFNAVG
DGIVLCKMINLSVPDTIDERTINKKKLTPFTIQENLNLALNSASAIGCHVVNIGAEDLKEGKPYLVLGLLWQVIKIGLFADIE
LSRNEALIALLREGESLEDLMKLSPEELLLRWANYHLENAGCNKIGNFSTDIKDSKAYYHLLEQVAPKGDEEGVPAVVID
MSGLREKDDIQRAECMLQQAERLGCRQFVTATDVVRGNPKLNLAFIANLFNRYPALHKPENQDIDWGALEGETREER
TFRNWMNSLGVNPRVNHLYSDLSDALVIFQLYEKIKVPVDWNRVNKPPYPKLGGNMKKLENCNYAVELGKNQAKFSL
VGIGGQDLNEGNRTLTLALIWQLMRRYTLNILEEIGGGQKVNDDIIVNWVNETLREAEKSSSISSFKDPKISTSLPVLDLID
AIQPGSINYDLLKTENLNDDEKLNNAKYAISMARKIGARVYALPEDLVEVNPKMVMTVFACLMGKGMKRV

Securinine soluble fraction/Spot 7/S100 Ca^{2+} binding protein A4/Mascot
MACPLEKALDVMVSTFHKYSGKEGDKFKLNKSELKELLTRELPSFLGKRTDEAAFQKLMSNLDSNRDNEVDFQEYCVF
LSCIAMMCNEFFEGFPDKQPRKK

Securinine soluble fraction/Spot 7/S100 Ca^{2+} binding protein A4/X!Hunter
MACPLEKALDVMVSTFHKYSGKEGDKFKLNKSELKELLTRELPSFLGKRTDEAAFQKLMSNLDSNRDNEVDFQEYCVFL
SCIAMMCNEFFEGFPDKQPRKK

not found by P3

Securinine soluble fraction/Spot 9/Hsp70/Mascot

MAKAAAIGIDLGTTYSCVGVFQHGKVEIIANDQGNRTTPSYVAFTDTERLIGDAAKNQVALNPQNTVFDAKRLIGRKF
GDPVVQSDMKHWPFQVINDGDKPKVQVSYKGETKAFYPEEISSMVLTKMKEIAEAYLGYPVTNAVITVPAYFNDSQR
QATKDAGVIAGLNVLRIINEPTAAAIAYGLDRTGKGERNVLIFDLGGGTFDVSILTIDDGIFEVKATAGDTHLGGEDFDN
RLVNHFVEEFKRKHKKDISQNKRAVRRLRTACERAKRTLSSSTQASLEIDSLFEGIDFYTSITRARFEELCSDLFRSTLEPVE
KALRDAKLDKAQIHDLVLVGGSTRIPKVQKLLQDFFNGRDLNKSINPDEAVAYGAAVQAAILMGDKSENVQDLLLLDV
APLSLGLETAGGVMTALIKRNSTIPTKQTQIFTTYSDNQPGVLIQVYEGERAMTKDNNLLGRFELSGIPPAPRGVPQIEV
TFDIDANGILNVTATDKSTGKANKITITNDKGRLSKEEIERMVQEAEKYKAEDEVQRERVSAKNALESYAFNMKSAVED
EGLKGKISEADKKKVLDKCQEVISWLDANTLAEKDEFEHKRKELEQVCNPIISGLYQGAGGPGPGGFGAQGPKGGSGS
GPTIEEVD

Securinine soluble fraction/Spot 9/Hsp70/X!Hunter

MSKGPAVGIDLGTTYSCVGVFQHGKVEIIANDQGNRTTPSYVAFTDTERLIGDAAKNQVAMNPTNTVFDAKRLIGRRF
DDAVVQSDMKHWPFMVVNDAGRPKVQVEYKGETKSFYPEEVSSMVLTKMKEIAEAYLGKTVTNAVVTVPAYFNDSQ
RQATKDAGTIAGLNVLRIINEPTAAAIAYGLDKKVGAERNVLIFDLGGGTFDVSILTIEDGIFEVKSTAGDTHLGGEDFDN
RMVNHFIAEFKRKHKKDISENKRAVRRLRTACERAKRTLSSSTQASIEIDSLYEGIDFYTSITRARFEELNADLFRGTLDPVE
KALRDAKLDKSQIHDIVLVGGSTRIPKIQKLLQDFFNGKELNKSINPDEAVAYGAAVQAAILSGDKSENVQDLLLLDVTPL
SLGIETAGGVMTVLIKRNTTIPTKQTQTFTTYSDNQPGVLIQVYEGERAMTKDNNLLGKFELTGIPPAPRGVPQIEVTFDI
DANGILNVSAVDKSTGKENKITITNDKGRLSKEDIERMVQEAEKYKAEDEKQRDKVSSKNSLESYAFNMKATVEDEKLQ
GKINDEDKQKILDKCNEIINWLDKNQTAEKEEFEHQQKELEKVCNPIITKLYQSAGGMPGGMPGGFPGGGAPPSGGAS
SGPTIEEVD

Securinine soluble fraction/Spot 9/Hsp70/X!Tandem P3

MAKAAAIGIDLGTTYSCVGVFQHGKVEIIANDQGNRTTPSYVAFTDTERLIGDAAKNQVALNPQNTVFDAKRLIGRKFG
DPVVQSDMKHWPFQVINDGDKPKVQVSYKGETKAFYPEEISSMVLTKMKEIAEAYLGYPVTNAVITVPAYFNDSQRQ
ATKDAGVIAGLNVLRIINEPTAAAIAYGLDRTGKGERNVLIFDLGGGTFDVSILTIDDGIFEVKATAGDTHLGGEDFDNRL
VNHFVEEFKRKHKKDISQNKRAVRRLRTACERAKRTLSSSTQASLEIDSLFEGIDFYTSITRARFEELCSDLFRSTLEPVEKAL
RDAKLDKAQIHDLVLVGGSTRIPKVQKLLQDFFNGRDLNKSINPDEAVAYGAAVQAAILMGDKSENVQDLLLLDVAPLS
LGLETAGGVMTALIKRNSTIPTKQTQIFTTYSDNQPGVLIQVYEGERAMTKDNNLLGRFELSGIPPAPRGVPQIEVTFDID
ANGILNVTATDKSTGKANKITITNDKGRLSKEEIERMVQEAEKYKAEDEVQRERVSAKNALESYAFNMKSAVEDEGLKG
KISEADKKKVLDKCQEVISWLDANTLAEKDEFEHKRKELEQVCNPIISGLYQGAGGPGPGGFGAQGPKGGSGSGPTIEE
VD

Securinine soluble fraction/Spot 10/L-plastin/Mascot

MARGSVSDEEMMELREAFAKVDTDGNGYISFNELNDLFKAACLPLPGYRVREITENLMATGDLDQDGRISFDEFIKIF
HGLKSTDVAKTFRKAINKKEGICAIGGTSEQSSVGTQHSYSEEEKYAFVNWINKALENDPDCRHVIPMNPNTNDLFNA
VGDGIVLCKMINLSVPDTIDERTINKKKLTPFTIQENLNLALNSASAIGCHVVNIGAEDLKEGKPYLVLGLLWQVIKIGLF
ADIELSRNEALIALLREGESLEDLMKLSPEELLLRWANYHLENAGCNKIGNFSTDIKDSKAYYHLLEQVAPKGDEEGVPA
VVIDMSGLREKDDIQRAECMLQQAERLGCRQFVTATDVVRGNPKLNLAFIANLFNRYPALHKPENQDIDWGALEGET
REERTFRNWMNSLGVNPRVNHLYSDLSDALVIFQLYEKIKVPVDWNRVNKPPYPKLGGNMKKLENCNYAVELGKNQ
AKFSLVGIGGQDLNEGNRTLTLALIWQLMRRYTLNILEEIGGGQKVNDDIIVNWVNETLREAEKSSSISSFKDPKISTSLP
VLDLIDAIQPGSINYDLLKTENLNDDEKLNNAKYAISMARKIGARVYALPEDLVEVNPKMVMTVFACLMGKGMKRV

Securinine soluble fraction/Spot 10/L-plastin/X!Hunter

MARGSVSDEEMMELREAFAKVDTDGNGYISFNELNDLFKAACLPLPGYRVREITENLMATGDLDQDGRISFDEFIKIFH
GLKSTDVAKTFRKAINKKEGICAIGGTSEQSSVGTQHSYSEEEKYAFVNWINKALENDPDCRHVIPMNPNTNDLFNAVG
DGIVLCKMINLSVPDTIDERTINKKKLTPFTIQENLNLALNSASAIGCHVVNIGAEDLKEGKPYLVLGLLWQVIKIGLFADIE
LSRNEALIALLREGESLEDLMKLSPEELLLRWANYHLENAGCNKIGNFSTDIKDSKAYYHLLEQVAPKGDEEGVPAVVID
MSGLREKDDIQRAECMLQQAERLGCRQFVTATDVVRGNPKLNLAFIANLFNRYPALHKPENQDIDWGALEGETREER
TFRNWMNSLGVNPRVNHLYSDLSDALVIFQLYEKIKVPVDWNRVNKPPYPKLGGNMKKLENCNYAVELGKNQAKFSL
VGIGGQDLNEGNRTLTLALIWQLMRRYTLNILEEIGGGQKVNDDIIVNWVNETLREAEKSSSISSFKDPKISTSLPVLDLID
AIQPGSINYDLLKTENLNDDEKLNNAKYAISMARKIGARVYALPEDLVEVNPKMVMTVFACLMGKGMKRV

Securinine soluble fraction/Spot 10/L-plastin/X!Tandem P3

MARGSVSDEEMMELREAFAKVDTDGNGYISFNELNDLFKAACLPLPGYRVREITENLMATGDLDQDGRISFDEFIKIFH
GLKSTDVAKTFRKAINKKEGICAIGGTSEQSSVGTQHSYSEEEKYAFVNWINKALENDPDCRHVIPMNPNTNDLFNAVG
DGIVLCKMINLSVPDTIDERTINKKKLTPFTIQENLNLALNSASAIGCHVVNIGAEDLKEGKPYLVLGLLWQVIKIGLFADIE
LSRNEALIALLREGESLEDLMKLSPEELLLRWANYHLENAGCNKIGNFSTDIKDSKAYYHLLEQVAPKGDEEGVPAVVID
MSGLREKDDIQRAECMLQQAERLGCRQFVTATDVVRGNPKLNLAFIANLFNRYPALHKPENQDIDWGALEGETREER
TFRNWMNSLGVNPRVNHLYSDLSDALVIFQLYEKIKVPVDWNRVNKPPYPKLGGNMKKLENCNYAVELGKNQAKFSL
VGIGGQDLNEGNRTLTLALIWQLMRRYTLNILEEIGGGQKVNDDIIVNWVNETLREAEKSSSISSFKDPKISTSLPVLDLID
AIQPGSINYDLLKTENLNDDEKLNNAKYAISMARKIGARVYALPEDLVEVNPKMVMTVFACLMGKGMKRV

Securinine soluble fraction/Spot 11/Thioredoxin 1/Mascot
MVKQIESKTAFQEALDAAGDKLVVVDFSATWCGPCKMIKPFFHSLSEKYSNVIFLEVDVDDCQDVASECEVKCMPTFQ
FFKKGQKVGEFSGANKEKLEATINELV

Securinine soluble fraction/Spot 11/Thioredoxin 1/X!Hunter
MVKQIESKTAFQEALDAAGDKLVVVDFSATWCGPCKMIKPFFHSLSEKYSNVIFLEVDVDDCQDVASECEVKCMPTFQ
FFKKGQKVGEFSGANKEKLEATINELV

Securinine soluble fraction/Spot 11/Thioredoxin 1/X!Tandem P3
MVKQIESKTAFQEALDAAGDKLVVVDFSATWCGPCKMIKPFFHSLSEKYSNVIFLEVDVDDCQDVASECEVKCMPTFQ
FFKKGQKVGEFSGANKEKLEATINELV

Securinine soluble fraction/Spot 12/Hsp70/Mascot
MSKGPAVGIDLGTTYSCVGVFQHGKVEIIANDQGNRTTPSYVAFTDTERLIGDAAKNQVAMNPTNTVFDAKRLIGRR
FDDAVVQSDMKHWPFMVVNDAGRPKVQVEYKGETKSFYPEEVSSMVLTKMKEIAEAYLGKTVTNAVVTVPAYFND
SQRQATKDAGTIAGLNVLRIINEPTAAAIAYGLDKKVGAERNVLIFDLGGGTFDVSILTIEDGIFEVKSTAGDTHLGGEDF
DNRMVNHFIAEFKRKHKKDISENKRAVRRLRTACERAKRTLSSSTQASIEIDSLYEGIDFYTSITRARFEELNADLFRGTLD
PVEKALRDAKLDKSQIHDIVLVGGSTRIPKIQKLLQDFFNGKELNKSINPDEAVAYGAAVQAAILSGDKSENVQDLLLLD
VTPLSLGIETAGGVMTVLIKRNTTIPTKQTQTFTTYSDNQPGVLIQVYEGERAMTKDNNLLGKFELTGIPPAPRGVPQIE
VTFDIDANGILNVSAVDKSTGKENKITITNDKGRLSKEDIERMVQEAEKYKAEDEKQRDKVSSKNSLESYAFNMKATVE
DEKLQGKINDEDKQKILDKCNEIINWLDKNQTAEKEEFEHQQKELEKVCNPIITKLYQSAGGMPGGMPGGFPGGGAP
PSGGASSGPTIEEVD

Securinine soluble fraction/Spot 12/Hsp70/X!Hunter
MSKGPAVGIDLGTTYSCVGVFQHGKVEIIANDQGNRTTPSYVAFTDTERLIGDAAKNQVAMNPTNTVFDAKRLIGRRF
DDAVVQSDMKHWPFMVVNDAGRPKVQVEYKGETKSFYPEEVSSMVLTKMKEIAEAYLGKTVTNAVVTVPAYFNDSQ
RQATKDAGTIAGLNVLRIINEPTAAAIAYGLDKKVGAERNVLIFDLGGGTFDVSILTIEDGIFEVKSTAGDTHLGGEDFDN
RMVNHFIAEFKRKHKKDISENKRAVRRLRTACERAKRTLSSSTQASIEIDSLYEGIDFYTSITRARFEELNADLFRGTLDPVE
KALRDAKLDKSQIHDIVLVGGSTRIPKIQKLLQDFFNGKELNKSINPDEAVAYGAAVQAAILSGDKSENVQDLLLLDVTPL
SLGIETAGGVMTVLIKRNTTIPTKQTQTFTTYSDNQPGVLIQVYEGERAMTKDNNLLGKFELTGIPPAPRGVPQIEVTFDI
DANGILNVSAVDKSTGKENKITITNDKGRLSKEDIERMVQEAEKYKAEDEKQRDKVSSKNSLESYAFNMKATVEDEKLQ
GKINDEDKQKILDKCNEIINWLDKNQTAEKEEFEHQQKELEKVCNPIITKLYQSAGGMPGGMPGGFPGGGAPPSGGAS
SGPTIEEVD

Securinine soluble fraction/Spot 12/Hsp70/X!Tandem P3
MSKGPAVGIDLGTTYSCVGVFQHGKVEIIANDQGNRTTPSYVAFTDTERLIGDAAKNQVAMNPTNTVFDAKRLIGRRF
DDAVVQSDMKHWPFMVVNDAGRPKVQVEYKGETKSFYPEEVSSMVLTKMKEIAEAYLGKTVTNAVVTVPAYFNDSQ
RQATKDAGTIAGLNVLRIINEPTAAAIAYGLDKKVGAERNVLIFDLGGGTFDVSILTIEDGIFEVKSTAGDTHLGGEDFDN
RMVNHFIAEFKRKHKKDISENKRAVRRLRTACERAKRTLSSSTQASIEIDSLYEGIDFYTSITRARFEELNADLFRGTLDPVE
KALRDAKLDKSQIHDIVLVGGSTRIPKIQKLLQDFFNGKELNKSINPDEAVAYGAAVQAAILSGDKSENVQDLLLLDVTPL
SLGIETAGGVMTVLIKRNTTIPTKQTQTFTTYSDNQPGVLIQVYEGERAMTKDNNLLGKFELTGIPPAPRGVPQIEVTFDI
DANGILNVSAVDKSTGKENKITITNDKGRLSKEDIERMVQEAEKYKAEDEKQRDKVSSKNSLESYAFNMKATVEDEKLQ
GKINDEDKQKILDKCNEIINWLDKNQTAEKEEFEHQQKELEKVCNPIITKLYQSAGGMPGGMPGGFPGGGAPPSGGAS
SGPTIEEVD

Securinine soluble fraction/Spot 13/L-plastin/Mascot

MARGSVSDEEMMELREAFAKVDTDGNGYISFNELNDLFKAACLPLPGYRVREITENLMATGDLDQDGRISFDEFIKIF
HGLKSTDVAKTFRKAINKKEGICAIGGTSEQSSVGTQHSYSEEEKYAFVNWINKALENDPDCRHVIPMNPNTNDLFNA
VGDGIVLCKMINLSVPDTIDERTINKKKLTPFTIQENLNLALNSASAIGCHVVNIGAEDLKEGKPYLVLGLLWQVIKIGLF
ADIELSRNEALIALLREGESLEDLMKLSPEELLLRWANYHLENAGCNKIGNFSTDIKDSKAYYHLLEQVAPKGDEEGVPA
VVIDMSGLREKDDIQRAECMLQQAERLGCRQFVTATDVVRGNPKLNLAFIANLFNRYPALHKPENQDIDWGALEGET
REERTFRNWMNSLGVNPRVNHLYSDLSDALVIFQLYEKIKVPVDWNRVNKPPYPKLGGNMKKLENCNYAVELGKNQ
AKFSLVGIGGQDLNEGNRTLTLALIWQLMRRYTLNILEEIGGGQKVNDDIIVNWVNETLREAEKSSSISSFKDPKISTSLP
VLDLIDAIQPGSINYDLLKTENLNDDEKLNNAKYAISMARKIGARVYALPEDLVEVNPKMVMTVFACLMGKGMKRV

Securinine soluble fraction/Spot 13/L-plastin/X!Hunter

MARGSVSDEEMMELREAFAKVDTDGNGYISFNELNDLFKAACLPLPGYRVREITENLMATGDLDQDGRISFDEFIKIFH
GLKSTDVAKTFRKAINKKEGICAIGGTSEQSSVGTQHSYSEEEKYAFVNWINKALENDPDCRHVIPMNPNTNDLFNAVG
DGIVLCKMINLSVPDTIDERTINKKKLTPFTIQENLNLALNSASAIGCHVVNIGAEDLKEGKPYLVLGLLWQVIKIGLFADIE
LSRNEALIALLREGESLEDLMKLSPEELLLRWANYHLENAGCNKIGNFSTDIKDSKAYYHLLEQVAPKGDEEGVPAVVID
MSGLREKDDIQRAECMLQQAERLGCRQFVTATDVVRGNPKLNLAFIANLFNRYPALHKPENQDIDWGALEGETREER
TFRNWMNSLGVNPRVNHLYSDLSDALVIFQLYEKIKVPVDWNRVNKPPYPKLGGNMKKLENCNYAVELGKNQAKFSL
VGIGGQDLNEGNRTLTLALIWQLMRRYTLNILEEIGGGQKVNDDIIVNWVNETLREAEKSSSISSFKDPKISTSLPVLDLID
AIQPGSINYDLLKTENLNDDEKLNNAKYAISMARKIGARVYALPEDLVEVNPKMVMTVFACLMGKGMKRV

Securinine soluble fraction/Spot 13/L-plastin/X!Tandem P3

MARGSVSDEEMMELREAFAKVDTDGNGYISFNELNDLFKAACLPLPGYRVREITENLMATGDLDQDGRISFDEFIKIFH
GLKSTDVAKTFRKAINKKEGICAIGGTSEQSSVGTQHSYSEEEKYAFVNWINKALENDPDCRHVIPMNPNTNDLFNAVG
DGIVLCKMINLSVPDTIDERTINKKKLTPFTIQENLNLALNSASAIGCHVVNIGAEDLKEGKPYLVLGLLWQVIKIGLFADIE
LSRNEALIALLREGESLEDLMKLSPEELLLRWANYHLENAGCNKIGNFSTDIKDSKAYYHLLEQVAPKGDEEGVPAVVID
MSGLREKDDIQRAECMLQQAERLGCRQFVTATDVVRGNPKLNLAFIANLFNRYPALHKPENQDIDWGALEGETREER
TFRNWMNSLGVNPRVNHLYSDLSDALVIFQLYEKIKVPVDWNRVNKPPYPKLGGNMKKLENCNYAVELGKNQAKFSL
VGIGGQDLNEGNRTLTLALIWQLMRRYTLNILEEIGGGQKVNDDIIVNWVNETLREAEKSSSISSFKDPKISTSLPVLDLID
AIQPGSINYDLLKTENLNDDEKLNNAKYAISMARKIGARVYALPEDLVEVNPKMVMTVFACLMGKGMKRV

Securinine soluble fraction/Spot 18/Hsp70/Mascot

MSKGPAVGIDLGTTYSCVGVFQHGKVEIIANDQGNRTTPSYVAFTDTERLIGDAAKNQVAMNPTNTVFDAKRLIGRR
FDDAVVQSDMKHWPFMVVNDAGRPKVQVEYKGETKSFYPEEVSSMVLTKMKEIAEAYLGKTVTNAVVTVPAYFND
SQRQATKDAGTIAGLNVLRIINEPTAAAIAYGLDKKVGAERNVLIFDLGGGTFDVSILTIEDGIFEVKSTAGDTHLGGEDF
DNRMVNHFIAEFKRKHKKDISENKRAVRRLRTACERAKRTLSSSTQASIEIDSLYEGIDFYTSITRARFEELNADLFRGTLD
PVEKALRDAKLDKSQIHDIVLVGGSTRIPKIQKLLQDFFNGKELNKSINPDEAVAYGAAVQAAILSGDKSENVQDLLLLD
VTPLSLGIETAGGVMTVLIKRNTTIPTKQTQTFTTYSDNQPGVLIQVYEGERAMTKDNNLLGKFELTGIPPAPRGVPQIE
VTFDIDANGILNVSAVDKSTGKENKITITNDKGRLSKEDIERMVQEAEKYKAEDEKQRDKVSSKNSLESYAFNMKATVE
DEKLQGKINDEDKQKILDKCNEIINWLDKNQTAEKEEFEHQQKELEKVCNPIITKLYQSAGGMPGGMPGGFPGGGAP
PSGGASSGPTIEEVD

Securinine soluble fraction/Spot 18/Hsp70/X!Hunter

MSKGPAVGIDLGTTYSCVGVFQHGKVEIIANDQGNRTTPSYVAFTDTERLIGDAAKNQVAMNPTNTVFDAKRLIGRRF
DDAVVQSDMKHWPFMVVNDAGRPKVQVEYKGETKSFYPEEVSSMVLTKMKEIAEAYLGKTVTNAVVTVPAYFNDSQ
RQATKDAGTIAGLNVLRIINEPTAAAIAYGLDKKVGAERNVLIFDLGGGTFDVSILTIEDGIFEVKSTAGDTHLGGEDFDN
RMVNHFIAEFKRKHKKDISENKRAVRRLRTACERAKRTLSSSTQASIEIDSLYEGIDFYTSITRARFEELNADLFRGTLDPVE
KALRDAKLDKSQIHDIVLVGGSTRIPKIQKLLQDFFNGKELNKSINPDEAVAYGAAVQAAILSGDKSENVQDLLLLDVTPL
SLGIETAGGVMTVLIKRNTTIPTKQTQTFTTYSDNQPGVLIQVYEGERAMTKDNNLLGKFELTGIPPAPRGVPQIEVTFDI
DANGILNVSAVDKSTGKENKITITNDKGRLSKEDIERMVQEAEKYKAEDEKQRDKVSSKNSLESYAFNMKATVEDEKLQ
GKINDEDKQKILDKCNEIINWLDKNQTAEKEEFEHQQKELEKVCNPIITKLYQSAGGMPGGMPGGFPGGGAPPSGGAS
SGPTIEEVD

Securinine soluble fraction/Spot 18/Hsp70/X!Tandem P3

MSKGPAVGIDLGTTYSCVGVFQHGKVEIIANDQGNRTTPSYVAFTDTERLIGDAAKNQVAMNPTNTVFDAKRLIGRRF
DDAVVQSDMKHWPFMVVNDAGRPKVQVEYKGETKSFYPEEVSSMVLTKMKEIAEAYLGKTVTNAVVTVPAYFNDSQ
RQATKDAGTIAGLNVLRIINEPTAAAIAYGLDKKVGAERNVLIFDLGGGTFDVSILTIEDGIFEVKSTAGDTHLGGEDFDN
RMVNHFIAEFKRKHKKDISENKRAVRRLRTACERAKRTLSSSTQASIEIDSLYEGIDFYTSITRARFEELNADLFRGTLDPVE
KALRDAKLDKSQIHDIVLVGGSTRIPKIQKLLQDFFNGKELNKSINPDEAVAYGAAVQAAILSGDKSENVQDLLLLDVTPL
SLGIETAGGVMTVLIKRNTTIPTKQTQTFTTYSDNQPGVLIQVYEGERAMTKDNNLLGKFELTGIPPAPRGVPQIEVTFDI
DANGILNVSAVDKSTGKENKITITNDKGRLSKEDIERMVQEAEKYKAEDEKQRDKVSSKNSLESYAFNMKATVEDEKLQ
GKINDEDKQKILDKCNEIINWLDKNQTAEKEEFEHQQKELEKVCNPIITKLYQSAGGMPGGMPGGFPGGGAPPSGGAS
SGPTIEEVD

Securinine soluble fraction/Spot 19/Hsp70/Mascot

MSKGPAVGIDLGTTYSCVGVFQHGKVEIIANDQGNRTTPSYVAFTDTERLIGDAAKNQVAMNPTNTVFDAKRLIGRR
FDDAVVQSDMKHWPFMVVNDAGRPKVQVEYKGETKSFYPEEVSSMVLTKMKEIAEAYLGKTVTNAVVTVPAYFND
SQRQATKDAGTIAGLNVLRIINEPTAAAIAYGLDKKVGAERNVLIFDLGGGTFDVSILTIEDGIFEVKSTAGDTHLGGEDF
DNRMVNHFIAEFKRKHKKDISENKRAVRRLRTACERAKRTLSSSTQASIEIDSLYEGIDFYTSITRARFEELNADLFRGTLD
PVEKALRDAKLDKSQIHDIVLVGGSTRIPKIQKLLQDFFNGKELNKSINPDEAVAYGAAVQAAILSGDKSENVQDLLLLD
VTPLSLGIETAGGVMTVLIKRNTTIPTKQTQTFTTYSDNQPGVLIQVYEGERAMTKDNNLLGKFELTGIPPAPRGVPQIE
VTFDIDANGILNVSAVDKSTGKENKITITNDKGRLSKEDIERMVQEAEKYKAEDEKQRDKVSSKNSLESYAFNMKATVE
DEKLQGKINDEDKQKILDKCNEIINWLDKNQTAEKEEFEHQQKELEKVCNPIITKLYQSAGGMPGGMPGGFPGGGAP
PSGGASSGPTIEEVD

Securinine soluble fraction/Spot 19/Hsp70/X!Hunter

MSKGPAVGIDLGTTYSCVGVFQHGKVEIIANDQGNRTTPSYVAFTDTERLIGDAAKNQVAMNPTNTVFDAKRLIGRRF
DDAVVQSDMKHWPFMVVNDAGRPKVQVEYKGETKSFYPEEVSSMVLTKMKEIAEAYLGKTVTNAVVTVPAYFNDSQ
RQATKDAGTIAGLNVLRIINEPTAAAIAYGLDKKVGAERNVLIFDLGGGTFDVSILTIEDGIFEVKSTAGDTHLGGEDFDN
RMVNHFIAEFKRKHKKDISENKRAVRRLRTACERAKRTLSSSTQASIEIDSLYEGIDFYTSITRARFEELNADLFRGTLDPVE
KALRDAKLDKSQIHDIVLVGGSTRIPKIQKLLQDFFNGKELNKSINPDEAVAYGAAVQAAILSGDKSENVQDLLLLDVTPL
SLGIETAGGVMTVLIKRNTTIPTKQTQTFTTYSDNQPGVLIQVYEGERAMTKDNNLLGKFELTGIPPAPRGVPQIEVTFDI
DANGILNVSAVDKSTGKENKITITNDKGRLSKEDIERMVQEAEKYKAEDEKQRDKVSSKNSLESYAFNMKATVEDEKLQ
GKINDEDKQKILDKCNEIINWLDKNQTAEKEEFEHQQKELEKVCNPIITKLYQSAGGMPGGMPGGFPGGGAPPSGGAS
SGPTIEEVD

Securinine soluble fraction/Spot 19/Hsp70/X!Tandem P3

MSKGPAVGIDLGTTYSCVGVFQHGKVEIIANDQGNRTTPSYVAFTDTERLIGDAAKNQVAMNPTNTVFDAKRLIGRRF
DDAVVQSDMKHWPFMVVNDAGRPKVQVEYKGETKSFYPEEVSSMVLTKMKEIAEAYLGKTVTNAVVTVPAYFNDSQ
RQATKDAGTIAGLNVLRIINEPTAAAIAYGLDKKVGAERNVLIFDLGGGTFDVSILTIEDGIFEVKSTAGDTHLGGEDFDN
RMVNHFIAEFKRKHKKDISENKRAVRRLRTACERAKRTLSSSTQASIEIDSLYEGIDFYTSITRARFEELNADLFRGTLDPVE
KALRDAKLDKSQIHDIVLVGGSTRIPKIQKLLQDFFNGKELNKSINPDEAVAYGAAVQAAILSGDKSENVQDLLLLDVTPL
SLGIETAGGVMTVLIKRNTTIPTKQTQTFTTYSDNQPGVLIQVYEGERAMTKDNNLLGKFELTGIPPAPRGVPQIEVTFDI
DANGILNVSAVDKSTGKENKITITNDKGRLSKEDIERMVQEAEKYKAEDEKQRDKVSSKNSLESYAFNMKATVEDEKLQ
GKINDEDKQKILDKCNEIINWLDKNQTAEKEEFEHQQKELEKVCNPIITKLYQSAGGMPGGMPGGFPGGGAPPSGGAS
SGPTIEEVD

Securinine soluble fraction/Spot 23/L-plastin/Mascot

MARGSVSDEEMMELREAFAKVDTDGNGYISFNELNDLFKAACLPLPGYRVREITENLMATGDLDQDGRISFDEFIKIF
HGLKSTDVAKTFRKAINKKEGICAIGGTSEQSSVGTQHSYSEEEKYAFVNWINKALENDPDCRHVIPMNPNTNDLFNA
VGDGIVLCKMINLSVPDTIDERTINKKKLTPFTIQENLNLALNSASAIGCHVVNIGAEDLKEGKPYLVLGLLWQVIKIGLF
ADIELSRNEALIALLREGESLEDLMKLSPEELLLRWANYHLENAGCNKIGNFSTDIKDSKAYYHLLEQVAPKGDEEGVPA
VVIDMSGLREKDDIQRAECMLQQAERLGCRQFVTATDVVRGNPKLNLAFIANLFNRYPALHKPENQDIDWGALEGET
REERTFRNWMNSLGVNPRVNHLYSDLSDALVIFQLYEKIKVPVDWNRVNKPPYPKLGGNMKKLENCNYAVELGKNQ
AKFSLVGIGGQDLNEGNRTLTLALIWQLMRRYTLNILEEIGGGQKVNDDIIVNWVNETLREAEKSSSISSFKDPKISTSLP
VLDLIDAIQPGSINYDLLKTENLNDDEKLNNAKYAISMARKIGARVYALPEDLVEVNPKMVMTVFACLMGKGMKRV

Securinine soluble fraction/Spot 23/L-plastin/X!Hunter

MARGSVSDEEMMELREAFAKVDTDGNGYISFNELNDLFKAACLPLPGYRVREITENLMATGDLDQDGRISFDEFIKIFH
GLKSTDVAKTFRKAINKKEGICAIGGTSEQSSVGTQHSYSEEEKYAFVNWINKALENDPDCRHVIPMNPNTNDLFNAVG
DGIVLCKMINLSVPDTIDERTINKKKLTPFTIQENLNLALNSASAIGCHVVNIGAEDLKEGKPYLVLGLLWQVIKIGLFADIE
LSRNEALIALLREGESLEDLMKLSPEELLLRWANYHLENAGCNKIGNFSTDIKDSKAYYHLLEQVAPKGDEEGVPAVVID
MSGLREKDDIQRAECMLQQAERLGCRQFVTATDVVRGNPKLNLAFIANLFNRYPALHKPENQDIDWGALEGETREER
TFRNWMNSLGVNPRVNHLYSDLSDALVIFQLYEKIKVPVDWNRVNKPPYPKLGGNMKKLENCNYAVELGKNQAKFSL
VGIGGQDLNEGNRTLTLALIWQLMRRYTLNILEEIGGGQKVNDDIIVNWVNETLREAEKSSSISSFKDPKISTSLPVLDLID
AIQPGSINYDLLKTENLNDDEKLNNAKYAISMARKIGARVYALPEDLVEVNPKMVMTVFACLMGKGMKRV

Securinine soluble fraction/Spot 23/L-plastin/X!Tandem P3

MARGSVSDEEMMELREAFAKVDTDGNGYISFNELNDLFKAACLPLPGYRVREITENLMATGDLDQDGRISFDEFIKIFH
GLKSTDVAKTFRKAINKKEGICAIGGTSEQSSVGTQHSYSEEEKYAFVNWINKALENDPDCRHVIPMNPNTNDLFNAVG
DGIVLCKMINLSVPDTIDERTINKKKLTPFTIQENLNLALNSASAIGCHVVNIGAEDLKEGKPYLVLGLLWQVIKIGLFADIE
LSRNEALIALLREGESLEDLMKLSPEELLLRWANYHLENAGCNKIGNFSTDIKDSKAYYHLLEQVAPKGDEEGVPAVVID
MSGLREKDDIQRAECMLQQAERLGCRQFVTATDVVRGNPKLNLAFIANLFNRYPALHKPENQDIDWGALEGETREER
TFRNWMNSLGVNPRVNHLYSDLSDALVIFQLYEKIKVPVDWNRVNKPPYPKLGGNMKKLENCNYAVELGKNQAKFSL
VGIGGQDLNEGNRTLTLALIWQLMRRYTLNILEEIGGGQKVNDDIIVNWVNETLREAEKSSSISSFKDPKISTSLPVLDLID
AIQPGSINYDLLKTENLNDDEKLNNAKYAISMARKIGARVYALPEDLVEVNPKMVMTVFACLMGKGMKRV

Securinine soluble fraction/Spot 27/SERPIN B1/Mascot
MEQLSSANTRFALDLFLALSENNPAGNIFISPFSISSAMAMVFLGTRGNTAAQLSKTFHFNTVEEVHSRFQSLNADINK
RGASYILKLANRLYGEKTYNFLPEFLVSTQKTYGADLASVDFQHASEDARKTINQWVKGQTEGKIPELLASGMVDNMT
KLVLVNAIYFKGNWKDKFMKEATTNAPFRLNKKDRKTVKMMYQKKKFAYGYIEDLKCRVLELPYQGEELSMVILLPDD
IEDESTGLKKIEEQLTLEKLHEWTKPENLDFIEVNVSLPRFKLEESYTLNSDLARLGVQDLFNSSKADLSGMSGARDIFISKI
VHKSFVEVNEEGTEAAAATAGIATFCMLMPEENFTADHPFLFFIRHNSSGSILFLGRFSSP

Securinine soluble fraction/Spot 27/SERPIN B1/X!Hunter
MEQLSSANTRFALDLFLALSENNPAGNIFISPFSISSAMAMVFLGTRGNTAAQLSKTFHFNTVEEVHSRFQSLNADINKR
GASYILKLANRLYGEKTYNFLPEFLVSTQKTYGADLASVDFQHASEDARKTINQWVKGQTEGKIPELLASGMVDNMTKL
VLVNAIYFKGNWKDKFMKEATTNAPFRLNKKDRKTVKMMYQKKKFAYGYIEDLKCRVLELPYQGEELSMVILLPDDIED
ESTGLKKIEEQLTLEKLHEWTKPENLDFIEVNVSLPRFKLEESYTLNSDLARLGVQDLFNSSKADLSGMSGARDIFISKIVHK
SFVEVNEEGTEAAAATAGIATFCMLMPEENFTADHPFLFFIRHNSSGSILFLGRFSSP

Securinine soluble fraction/Spot 27/SERPIN B1/X!Tandem P3
MEQLSSANTRFALDLFLALSENNPAGNIFISPFSISSAMAMVFLGTRGNTAAQLSKTFHFNTVEEVHSRFQSLNADINKR
GASYILKLANRLYGEKTYNFLPEFLVSTQKTYGADLASVDFQHASEDARKTINQWVKGQTEGKIPELLASGMVDNMTKL
VLVNAIYFKGNWKDKFMKEATTNAPFRLNKKDRKTVKMMYQKKKFAYGYIEDLKCRVLELPYQGEELSMVILLPDDIED
ESTGLKKIEEQLTLEKLHEWTKPENLDFIEVNVSLPRFKLEESYTLNSDLARLGVQDLFNSSKADLSGMSGARDIFISKIVHK
SFVEVNEEGTEAAAATAGIATFCMLMPEENFTADHPFLFFIRHNSSGSILFLGRFSSP

Securinine soluble fraction/Spot 73/Hsp90/Mascot

MPEEVHHGEEEVETFAFQAEIAQLMSLIINTFYSNKEIFLRELISNASDALDKIRYESLTDPSKLDSGKELKIDIIPNPQERTL
TLVDTGIGMTKADLINNLGTIAKSGTKAFMEALQAGADISMIGQFGVGFYSAYLVAEKVVVITKHNDDEQYAWESSA
GGSFTVRADHGEPIGRGTKVILHLKEDQTEYLEERRVKEVVKKHSQFIGYPITLYLEKEREKEISDDEAEEEKGEKEEEDKD
DEEKPKIEDVGSDEEDDSGKDKKKKTKKIKEKYIDQEELNKTKPIWTRNPDDITQEEYGEFYKSLTNDWEDHLAVKHFS
VEGQLEFRALLFIPRRAPFDLFENKKKKNNIKLYVRRVFIMDSCDELIPEYLNFIRGVVDSEDLPLNISREMLQQSKILKAIR
KNIVKKCLELFSELAEDKENYKKFYEAFSKNLKLGIHEDSTNRRRLSELLRYHTSQSGDEMTSLSEYVSRMKETQKSIYYIT
GESKEQVANSAFVERVRKRGFEVVYMTEPIDEYCVQQLKEFDGKSLVSVTKEGLELPEDEEEKKKMEESKAKFENLCKL
MKEILDKKVEKVTISNRLVSSPCCIVTSTYGWTANMERIMKAQALRDNSTMGYMMAKKHLEINPDHPIVETLRQKAE
ADKNDKAVKDLVVLLFETALLSSGFSLEDPQTHSNRIYRMIKLGLGIDEDEVAAEEPNAAVPDEIPLSRAMRMRLAWK
KSIRLGVHSWKTCALV

Securinine soluble fraction/Spot 73/Hsp90/X!Hunter

MPEEVHHGEEEVETFAFQAEIAQLMSLIINTFYSNKEIFLRELISNASDALDKIRYESLTDPSKLDSGKELKIDIIPNPQERTL
TLVDTGIGMTKADLINNLGTIAKSGTKAFMEALQAGADISMIGQFGVGFYSAYLVAEKVVVITKHNDDEQYAWESSAG
GSFTVRADHGEPIGRGTKVILHLKEDQTEYLEERRVKEVVKKHSQFIGYPITLYLEKEREKEISDDEAEEEKGEKEEEDKDD
EEKPKIEDVGSDEEDDSGKDKKKKTKKIKEKYIDQEELNKTKPIWTRNPDDITQEEYGEFYKSLTNDWEDHLAVKHFSVE
GQLEFRALLFIPRRAPFDLFENKKKKNNIKLYVRRVFIMDSCDELIPEYLNFIRGVVDSEDLPLNISREMLQQSKILKVIRKNI
VKKCLELFSELAEDKENYKKFYEAFSKNLKLGIHEDSTNRRRLSELLRYHTSQSGDEMTSLSEYVSRMKETQKSIYYITGESK
EQVANSAFVERVRKRGFEVVYMTEPIDEYCVQQLKEFDGKSLVSVTKEGLELPEDEEEKKKMEESKAKFENLCKLMKEIL
DKKVEKVTISNRLVSSPCCIVTSTYGWTANMERIMKAQALRDNSTMGYMMAKKHLEINPDHPIVETLRQKAEADKND
KAVKDLVVLLFETALLSSGFSLEDPQTHSNRIYRMIKLGLGIDEDEVAAEEPNAAVPDEIPPLEGDEDASRMEEVD

Securinine soluble fraction/Spot 73/Hsp90/X!Tandem P3

MPEEVHHGEEEVETFAFQAEIAQLMSLIINTFYSNKEIFLRELISNASDALDKIRYESLTDPSKLDSGKELKIDIIPNPQERTL
TLVDTGIGMTKADLINNLGTIAKSGTKAFMEALQAGADISMIGQFGVGFYSAYLVAEKVVVITKHNDDEQYAWESSAG
GSFTVRADHGEPIGRGTKVILHLKEDQTEYLEERRVKEVVKKHSQFIGYPITLYLEKEREKEISDDEAEEEKGEKEEEDKDD
EEKPKIEDVGSDEEDDSGKDKKKKTKKIKEKYIDQEELNKTKPIWTRNPDDITQEEYGEFYKSLTNDWEDHLAVKHFSVE
GQLEFRALLFIPRRAPFDLFENKKKKNNIKLYVRRVFIMDSCDELIPEYLNFIRGVVDSEDLPLNISREMLQQSKILKVIRKNI
VKKCLELFSELAEDKENYKKFYEAFSKNLKLGIHEDSTNRRRLSELLRYHTSQSGDEMTSLSEYVSRMKETQKSIYYITGESK
EQVANSAFVERVRKRGFEVVYMTEPIDEYCVQQLKEFDGKSLVSVTKEGLELPEDEEEKKKMEESKAKFENLCKLMKEIL
DKKVEKVTISNRLVSSPCCIVTSTYGWTANMERIMKAQALRDNSTMGYMMAKKHLEINPDHPIVETLRQKAEADKND
KAVKDLVVLLFETALLSSGFSLEDPQTHSNRIYRMIKLGLGIDEDEVAAEEPNAAVPDEIPPLEGDEDASRMEEVD

Securinine soluble fraction/Spot 83/Actin/Mascot

MDDDIAALVVDNGSGMCKAGFAGDDAPRAVFPSIVGRPRHQGVMVGMGQKDSYVGDEAQSKRGILTLKYPIEHGI
VTNWDDMEKIWHHTFYNELRVAPEEHPVLLTEAPLNPKANREKMTQIMFETFNTPAMYVAIQAMLSLYASGRTTGI
VMDSGDGVTHTVPIYEGYALPHAILRLDLAGRDLTDYLMKILTERGYSFTTTAEREIVRDIKEKLCYVALDFEQEMATAA
SSSSLEKSYELPDGQVITIGNERFRCPEALFQPSFLGMESCGIHETTFNSIMKCDVDIRKDLYDNTVLSGGTTMYPGIAD
RMQKEITALAPSTMKIKIIAPPERKYSVWIGGSILASLSTFQQMWISKQEYDESGPSIVHRKCF

Securinine soluble fraction/Spot 83/Actin/X!Hunter

MEEEIAALVIDNGSGMCKAGFAGDDAPRAVFPSIVGRPRHQGVMVGMGQKDSYVGDEAQSKRGILTLKYPIEHGIVT
NWDDMEKIWHHTFYNELRVAPEEHPVLLTEAPLNPKANREKMTQIMFETFNTPAMYVAIQAVLSLYASGRTTGIVMD
SGDGVTHTVPIYEGYALPHAILRLDLAGRDLTDYLMKILTERGYSFTTTAEREIVRDIKEKLCYVALDFEQEMATAASSSL
EKSYELPDGQVITIGNERFRCPEALFQPSFLGMESCGIHETTFNSIMKCDVDIRKDLYANTVLSGGTTMYPGIADRMQKE
ITALAPSTMKIKIIAPPERKYSVWIGGSILASLSTFQQMWISKQEYDESGPSIVHRKCF

Securinine soluble fraction/Spot 83/Actin/X!Tandem P3

MEEEIAALVIDNGSGMCKAGFAGDDAPRAVFPSIVGRPRHQGVMVGMGQKDSYVGDEAQSKRGILTLKYPIEHGIVT
NWDDMEKIWHHTFYNELRVAPEEHPVLLTEAPLNPKANREKMTQIMFETFNTPAMYVAIQAVLSLYASGRTTGIVMD
SGDGVTHTVPIYEGYALPHAILRLDLAGRDLTDYLMKILTERGYSFTTTAEREIVRDIKEKLCYVALDFEQEMATAASSSL
EKSYELPDGQVITIGNERFRCPEALFQPSFLGMESCGIHETTFNSIMKCDVDIRKDLYANTVLSGGTTMYPGIADRMQKE
ITALAPSTMKIKIIAPPERKYSVWIGGSILASLSTFQQMWISKQEYDESGPSIVHRKCF

Securinine soluble fraction/Spot 89/Hsp60/Mascot

MLRLPTVFRQMRPVSRVLAPHLTRAYAKDVKFGADARALMLQGVDLLADAVAVTMGPKGRTVIIEQSWGSPKVTKD
GVTVAKSIDLKDKYKNIGAKLVQDVANNTNEEAGDGTTTATVLARSIAKEGFEKISKGANPVEIRRGVMLAVDAVIAEL
KKQSKPVTTPEEIAQVATISANGDKEIGNIISDAMKKVGRKGVITVKDGKTLNDELEIIEGMKFDRGYISPYFINTSKGQK
CEFQDAYVLLSEKKISSIQSIVPALEIANAHRKPLVIIAEDVDGEALSTLVLNRLKVGLQVVAVKAPGFGDNRKNQLKDM
AIATGGAVFGEEGLTLNLEDVQPHDLGKVGEVIVTKDDAMLLKGKGDKAQIEKRIQEIIEQLDVTTSEYEKEKLNERLAK
LSDGVAVLKVGGTSDVEVNEKKDRVTDALNATRAAVEEGIVLGGGCALLRCIPALDSLTPANEDQKIGIEIIKRTLKIPA
MTIAKNAGVEGSLIVEKIMQSSSEVGYDAMAGDFVNMVEKGIIDPTKVVRTALLDAAGVASLLTTAEVVVTEIPKEEKD
PGMGAMGGMGGGMGGGMF

Securinine soluble fraction/Spot 89/Hsp60/X!Hunter

MLRLPTVFRQMRPVSRVLAPHLTRAYAKDVKFGADARALMLQGVDLLADAVAVTMGPKGRTVIIEQSWGSPKVTKD
GVTVAKSIDLKDKYKNIGAKLVQDVANNTNEEAGDGTTTATVLARSIAKEGFEKISKGANPVEIRRGVMLAVDAVIAELK
KQSKPVTTPEEIAQVATISANGDKEIGNIISDAMKKVGRKGVITVKDGKTLNDELEIIEGMKFDRGYISPYFINTSKGQKCE
FQDAYVLLSEKKISSIQSIVPALEIANAHRKPLVIIAEDVDGEALSTLVLNRLKVGLQVVAVKAPGFGDNRKNQLKDMAIA
TGGAVFGEEGLTLNLEDVQPHDLGKVGEVIVTKDDAMLLKGKGDKAQIEKRIQEIIEQLDVTTSEYEKEKLNERLAKLSD
GVAVLKVGGTSDVEVNEKKDRVTDALNATRAAVEEGIVLGGGCALLRCIPALDSLTPANEDQKIGIEIIKRTLKIPAMTIAK
NAGVEGSLIVEKIMQSSSEVGYDAMAGDFVNMVEKGIIDPTKVVRTALLDAAGVASLLTTAEVVVTEIPKEEKDPGMG
AMGGMGGGMGGGMF

Securinine soluble fraction/Spot 89/Hsp60/X!Tandem P3

MLRLPTVFRQMRPVSRVLAPHLTRAYAKDVKFGADARALMLQGVDLLADAVAVTMGPKGRTVIIEQSWGSPKVTKD
GVTVAKSIDLKDKYKNIGAKLVQDVANNTNEEAGDGTTTATVLARSIAKEGFEKISKGANPVEIRRGVMLAVDAVIAELK
KQSKPVTTPEEIAQVATISANGDKEIGNIISDAMKKVGRKGVITVKDGKTLNDELEIIEGMKFDRGYISPYFINTSKGQKCE
FQDAYVLLSEKKISSIQSIVPALEIANAHRKPLVIIAEDVDGEALSTLVLNRLKVGLQVVAVKAPGFGDNRKNQLKDMAIA
TGGAVFGEEGLTLNLEDVQPHDLGKVGEVIVTKDDAMLLKGKGDKAQIEKRIQEIIEQLDVTTSEYEKEKLNERLAKLSD
GVAVLKVGGTSDVEVNEKKDRVTDALNATRAAVEEGIVLGGGCALLRCIPALDSLTPANEDQKIGIEIIKRTLKIPAMTIAK
NAGVEGSLIVEKIMQSSSEVGYDAMAGDFVNMVEKGIIDPTKVVRTALLDAAGVASLLTTAEVVVTEIPKEEKDPGMG
AMGGMGGGMGGGMF

Securinine soluble fraction/Spot 96/Inosine 5' monophosphate dehydrogenase/Mascot
MADYLISGGTSYVPDDGLTAQQLFNCGDGLTYNDFLILPGYIDFTADQVDLTSALTKKITLKTPLVSSPMDTVTEAGMA
IAMALTGGIGFIHHNCTPEFQANEVRKVKKYEQGFITDPVVLSPKDRVRDVFEAKARHGFCGIPITDTGRMGSRLVGIIS
SRDIDFLKEEEHDCFLEEIMTKREDLVVAPAGITLKEANEILQRSKKGKLPIVNEDDELVAIIARTDLKKNRDYPLASKDAK
KQLLCGAAIGTHEDDKYRLDLLAQAGVDVVVLDSSQGNSIFQINMIKYIKDKYPNLQVIGGNVVTAAQAKNLIDAGVD
ALRVGMGSGSICITQEVLACGRPQATAVYKVSEYARRFGVPVIADGGIQNVGHIAKALALGASTVMMGSLLAATTEAP
GEYFFSDGIRLKKYRGMGSLDAMDKHLSSQNRYFSEADKIKVAQGVSGAVQDKGSIHKFVPYLIAGIQHSCQDIGAKSL
TQVRAMMYSGELKFEKRTSSAQVEGGVHSLHSYEKRLF

Securinine soluble fraction/Spot 96/Inosine 5' monophosphate dehydrogenase/X!Hunter
MADYLISGGTSYVPDDGLTAQQLFNCGDGLTYNDFLILPGYIDFTADQVDLTSALTKKITLKTPLVSSPMDTVTEAGMAI
AMALTGGIGFIHHNCTPEFQANEVRKVKKYEQGFITDPVVLSPKDRVRDVFEAKARHGFCGIPITDTGRMGSRLVGIISS
RDIDFLKEEEHDCFLEEIMTKREDLVVAPAGITLKEANEILQRSKKGKLPIVNEDDELVAIIARTDLKKNRDYPLASKDAKK
QLLCGAAIGTHEDDKYRLDLLAQAGVDVVVLDSSQGNSIFQINMIKYIKDKYPNLQVIGGNVVTAAQAKNLIDAGVDAL
RVGMGSGSICITQEVLACGRPQATAVYKVSEYARRFGVPVIADGGIQNVGHIAKALALGASTVMMGSLLAATTEAPGE
YFFSDGIRLKKYRGMGSLDAMDKHLSSQNRYFSEADKIKVAQGVSGAVQDKGSIHKFVPYLIAGIQHSCQDIGAKSLTQ
VRAMMYSGELKFEKRTSSAQVEGGVHSLHSYEKRLF

Securinine soluble fraction/Spot 96/Inosine 5' monophosphate dehydrogenase/X!Tandem P3
MADYLISGGTSYVPDDGLTAQQLFNCGDGLTYNDFLILPGYIDFTADQVDLTSALTKKITLKTPLVSSPMDTVTEAGMAI
AMALTGGIGFIHHNCTPEFQANEVRKVKKYEQGFITDPVVLSPKDRVRDVFEAKARHGFCGIPITDTGRMGSRLVGIISS
RDIDFLKEEEHDCFLEEIMTKREDLVVAPAGITLKEANEILQRSKKGKLPIVNEDDELVAIIARTDLKKNRDYPLASKDAKK
QLLCGAAIGTHEDDKYRLDLLAQAGVDVVVLDSSQGNSIFQINMIKYIKDKYPNLQVIGGNVVTAAQAKNLIDAGVDAL
RVGMGSGSICITQEVLACGRPQATAVYKVSEYARRFGVPVIADGGIQNVGHIAKALALGASTVMMGSLLAATTEAPGE
YFFSDGIRLKKYRGMGSLDAMDKHLSSQNRYFSEADKIKVAQGVSGAVQDKGSIHKFVPYLIAGIQHSCQDIGAKSLTQ
VRAMMYSGELKFEKRTSSAQVEGGVHSLHSYEKRLF

Securinine membrane fraction/Spot 6/Hsp70/Mascot
MSKGPAVGIDLGTTYSCVGVFQHGKVEIIANDQGNRTTPSYVAFTDTERLIGDAAKNQVAMNPTNTVFDAKRLIGRR
FDDAVVQSDMKHWPFMVVNDAGRPKVQVEYKGETKSFYPEEVSSMVLTKMKEIAEAYLGKTVTNAVVTVPAYFND
SQRQATKDAGTIAGLNVLRIINEPTAAAIAYGLDKKVGAERNVLIFDLGGGTFDVSILTIEDGIFEVKSTAGDTHLGGEDF
DNRMVNHFIAEFKRKHKKDISENKRAVRRLRTACERAKRTLSSSTQASIEIDSLYEGIDFYTSITRARFEELNADLFRGTLD
PVEKALRDAKLDKSQIHDIVLVGGSTRIPKIQKLLQDFFNGKELNKSINPDEAVAYGAAVQAAILSGDKSENVQDLLLLD
VTPLSLGIETAGGVMTVLIKRNTTIPTKQTQTFTTYSDNQPGVLIQVYEGERAMTKDNNLLGKFELTGIPPAPRGVPQIE
VTFDIDANGILNVSAVDKSTGKENKITITNDKGRLSKEDIERMVQEAEKYKAEDEKQRDKVSSKNSLESYAFNMKATVE
DEKLQGKINDEDKQKILDKCNEIINWLDKNQTAEKEEFEHQQKELEKVCNPIITKLYQSAGGMPGGMPGGFPGGGAP
PSGGASSGPTIEEVD

Securinine membrane fraction/Spot 6/Hsp70/X!Hunter
MSKGPAVGIDLGTTYSCVGVFQHGKVEIIANDQGNRTTPSYVAFTDTERLIGDAAKNQVAMNPTNTVFDAKRLIGRRF
DDAVVQSDMKHWPFMVVNDAGRPKVQVEYKGETKSFYPEEVSSMVLTKMKEIAEAYLGKTVTNAVVTVPAYFNDSQ
RQATKDAGTIAGLNVLRIINEPTAAAIAYGLDKKVGAERNVLIFDLGGGTFDVSILTIEDGIFEVKSTAGDTHLGGEDFDN
RMVNHFIAEFKRKHKKDISENKRAVRRLRTACERAKRTLSSSTQASIEIDSLYEGIDFYTSITRARFEELNADLFRGTLDPVE
KALRDAKLDKSQIHDIVLVGGSTRIPKIQKLLQDFFNGKELNKSINPDEAVAYGAAVQAAILSGDKSENVQDLLLLDVTPL
SLGIETAGGVMTVLIKRNTTIPTKQTQTFTTYSDNQPGVLIQVYEGERAMTKDNNLLGKFELTGIPPAPRGVPQIEVTFDI
DANGILNVSAVDKSTGKENKITITNDKGRLSKEDIERMVQEAEKYKAEDEKQRDKVSSKNSLESYAFNMKATVEDEKLQ
GKINDEDKQKILDKCNEIINWLDKNQTAEKEEFEHQQKELEKVCNPIITKLYQSAGGMPGGMPGGFPGGGAPPSGGAS
SGPTIEEVD

Securinine membrane fraction/Spot 6/Hsp70/X!Tandem P3
MSKGPAVGIDLGTTYSCVGVFQHGKVEIIANDQGNRTTPSYVAFTDTERLIGDAAKNQVAMNPTNTVFDAKRLIGRRF
DDAVVQSDMKHWPFMVVNDAGRPKVQVEYKGETKSFYPEEVSSMVLTKMKEIAEAYLGKTVTNAVVTVPAYFNDSQ
RQATKDAGTIAGLNVLRIINEPTAAAIAYGLDKKVGAERNVLIFDLGGGTFDVSILTIEDGIFEVKSTAGDTHLGGEDFDN
RMVNHFIAEFKRKHKKDISENKRAVRRLRTACERAKRTLSSSTQASIEIDSLYEGIDFYTSITRARFEELNADLFRGTLDPVE
KALRDAKLDKSQIHDIVLVGGSTRIPKIQKLLQDFFNGKELNKSINPDEAVAYGAAVQAAILSGDKSENVQDLLLLDVTPL
SLGIETAGGVMTVLIKRNTTIPTKQTQTFTTYSDNQPGVLIQVYEGERAMTKDNNLLGKFELTGIPPAPRGVPQIEVTFDI
DANGILNVSAVDKSTGKENKITITNDKGRLSKEDIERMVQEAEKYKAEDEKQRDKVSSKNSLESYAFNMKATVEDEKLQ
GKINDEDKQKILDKCNEIINWLDKNQTAEKEEFEHQQKELEKVCNPIITKLYQSAGGMPGGMPGGFPGGGAPPSGGAS
SGPTIEEVD

Securinine membrane fraction/Spot 52/Actin/Mascot
MDDDIAALVVDNGSGMCKAGFAGDDAPRAVFPSIVGRPRHQGVMVGMGQKDSYVGDEAQSKRGILTLKYPIEHGI
VTNWDDMEKIWHHTFYNELRVAPEEHPVLLTEAPLNPKANREKMTQIMFETFNTPAMYVAIQAVLSLYASGRTTGIV
MDSGDGVTHTVPIYEGYALPHAILRLDLAGRDLTDYLMKILTERGYSFTTTAEREIVRDIKEKLCYVALDFEQEMATAAS
SSSLEKSYELPDGQVITIGNERFRCPEALFQPSFLGMESCGIHETTFNSIMKCDVDIRKDLYANTVLSGGTTMYPGIADR
MQKEITALAPSTMKIKIIAPPERKYSVWIGGSILASLSTFQQMWISKQEYDESGPSIVHRKCF

Securinine membrane fraction/Spot 52/Actin/X!Hunter
MEEEIAALVIDNGSGMCKAGFAGDDAPRAVFPSIVGRPRHQGVMVGMGQKDSYVGDEAQSKRGILTLKYPIEHGIVT
NWDDMEKIWHHTFYNELRVAPEEHPVLLTEAPLNPKANREKMTQIMFETFNTPAMYVAIQAVLSLYASGRTTGIVMD
SGDGVTHTVPIYEGYALPHAILRLDLAGRDLTDYLMKILTERGYSFTTTAEREIVRDIKEKLCYVALDFEQEMATAASSSSL
EKSYELPDGQVITIGNERFRCPEALFQPSFLGMESCGIHETTFNSIMKCDVDIRKDLYANTVLSGGTTMYPGIADRMQKE
ITALAPSTMKIKIIAPPERKYSVWIGGSILASLSTFQQMWISKQEYDESGPSIVHRKCF

Securinine membrane fraction/Spot 52/Actin/X!Tandem P3
MDDDIAALVVDNGSGMCKAGFAGDDAPRAVFPSIVGRPRHQGVMVGMGQKDSYVGDEAQSKRGILTLKYPIEHGIV
TNWDDMEKIWHHTFYNELRVAPEEHPVLLTEAPLNPKANREKMTQIMFETFNTPAMYVAIQAVL
SLYASGRTTGIVMDSGDGVTHTVPIYEGYALPHAILRLDLAGRDLTDYLMKILTERGYSFTTTAEREIVR
DIKEKLCYVALDFEQEMATAASSSSLEKSYELPDGQVITIGNERFRCPEALFQPSFLGMESCGIHETTFN
SIMKCDVDIRKDLYANTVLSGGTTMYPGIADRMQKEITALAPSTMKIKIIAPPERKYSVWIGGSILASLS
TFQQMWISKQEYDESGPSIVHRKCF
