# Supplementary material for: Proteomic and Systems Biology Analysis of Monocytes Exposed to Securinine, a GABAA Receptor Antagonist and Immune Adjuvant
Source: PLoS One. 2012 Sep 13;7(9):e41278. doi: 10.1371/journal.pone.0041278 (PMC3441550; doi:10.1371/journal.pone.0041278)

Supplemental figure 1

Supplemental figure 1: Securinine protein interaction/activity network model

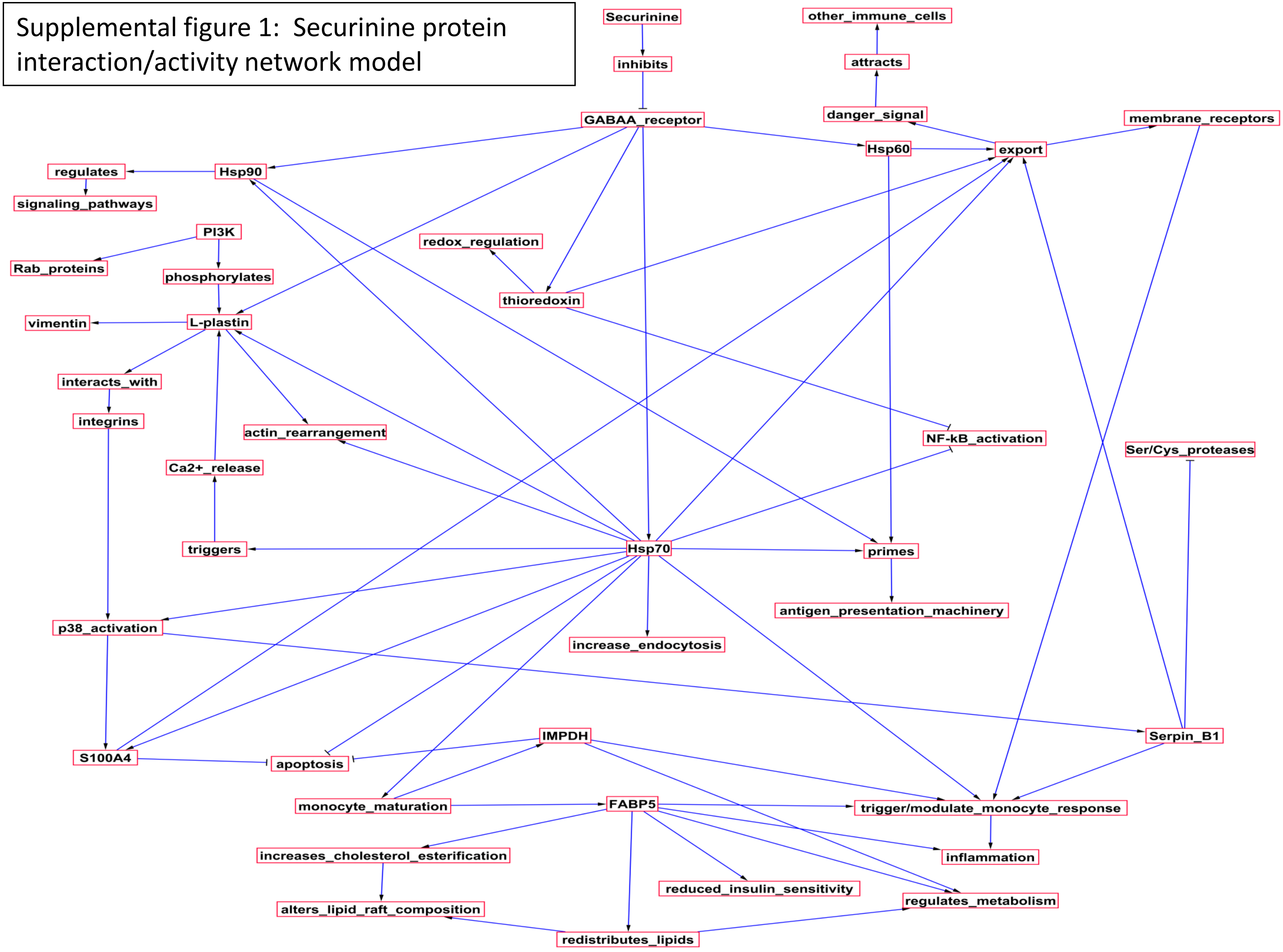

Supplement: Figure S1 — Systems biology network model proposed for Monomac I cellular response to stimulation by Securinine. Graph of the network model generated by the manual curation of the literature. See text for details of this model. (PDF) [file pone.0041278.s003.pdf]
