# Supplementary figures and images for: Proteomic and Systems Biology Analysis of Monocytes Exposed to Securinine, a GABAA Receptor Antagonist and Immune Adjuvant
Source: PLoS One. 2012 Sep 13;7(9):e41278. doi: 10.1371/journal.pone.0041278 (PMC3441550; doi:10.1371/journal.pone.0041278)

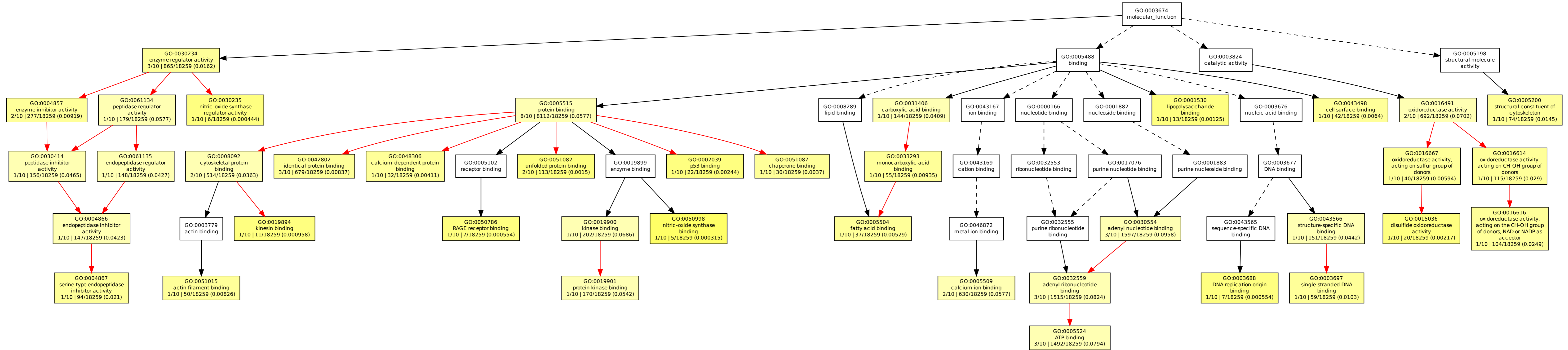

Supplement: Figure S2 — GOEAST model showing the molecular functions of the differentially expressed proteins from Securinine stimulated Monomac I cells. In this image, a sample of the data from GOEAST searches are displayed. The data displayed shows a search of GO terms related to molecular functions for the differentially expressed proteins in the present investigation. A) complete graph from GOEAST showing various connections. Yellow squares indicate that at least one accession number from supplemental Table 1 was identified for that GO term. White squares indicate that no accession numbers were found. Red arrows go from one detected accession number to another (can be the same term for more than one function), while the black arrows extend from a GO term which no accession numbers were associated with. (PDF) [file pone.0041278.s004.pdf]
